# The impact of oxygen supply flow rates on the distribution of aerosols between the patient and the surgeon during lung intubation

**DOI:** 10.1007/s44189-022-00006-4

**Published:** 2022-06-28

**Authors:** Tee Lin, Omid Ali Zargar, Ming-Hsuan Hu, Chung-Chun Wang, Shih-Cheng Hu, Graham Leggett

**Affiliations:** 1 Department of Energy and Refrigerating Air-conditioning Engineering, National Taipei University of Technology, No. 1, Section 3, Zhongxiao East Road, Da’an District, Taipei, Taiwan, People’s Republic of China; 2 LI-COR Biosciences, Lincoln, NE USA

**Keywords:** COVID-19, Intubation therapy, Particle image velocimetry (PIV), Flow field visualization, PAO

## Abstract

The outbreak of COVID-19 has caused a worldwide pandemic. The widespread infection of the medical staff has caused great attention from all quarters of society. There is a particular concern when considering intubation treatment in the emergency operating room, where a significant amount of virus droplets are typically spread within the room, exposing the medical staff to a high risk of infection. Hence, there is currently a pressing need to develop an effective protection mechanism for the medical staff to prevent them from being infected during routine work. In order to understand the spread of droplets and aerosols when different oxygen supply devices are used for intubation therapy, this study uses particle image velocimetry (PIV) technology to analyze the airflow distribution between the medical staff and the patient. In the experiment, a simple version of the respirator was established to reproduce the breathing of human lungs. This model used oil to create smoke as a tracer aerosol, then a high-sensitivity camera was used to record the scattering light from this smoke (which is irradiated by the green laser sheet). Ultimately, after applying post-processing techniques, the airflow distribution is analyzed. PAO aerosol is the primary aerosol source in this experiment, and it is used to quantify the patient’s breathing; the concentration of PAO aerosol was measured at three different points: head, trunk, and feet. In addition, flow field visualization can effectively present the flow field distribution of the entire operating room; also, the results can be mutually verified with the PAO concentration measurement results. Aerosol concentrations were measured for six different oxygen supply devices with various tidal volumes of the artificial respirator, and the results were ranked from high to low concentrations for different oxygen supply devices and their operational oxygen supply flowrates: HFNC (70 l/min) > CPAP (40 l/min) > HFNC (30 l/min) > nasal cannula (15 l/min) > NRM (15 l/min) > VAPOX (28 l/min).

## Highlights

• The effect of different oxygen supply on the droplets exhaled by patients during lung intubation treatment were examined.

• the PAO aerosols were released at the mouth of the manikin to simulate the aerosols produced during intubation processes.

• variations of concentrations of PAO aerosols and the flow fields were analyzed.

• The results could be beneficial to minimize the impact of different oxygen supply equipment on medical staff.

## Introduction

The COVID-19 virus can attach to aerosols in the air. By inhaling these particles that are lighter than droplets, humans can be infected with COVID-19 [according to reports from the US center for disease control and prevention (CDC)]. This statement confirms that COVID-19 is an “airborne” disease. Research from the Massachusetts Institute of Technology (MIT) [1, 2] also indicates that in an indoor environment, the amount of the virus to which a healthy individual is exposed from an infected person is the same under the social distance of 1.8 m and 18 m, which means keeping the social distance further than 1.8 m is less relevant in lowering the infection risk. The spread of coronavirus is more related to issues such as the indoor population, mask-wearing, and ventilation. According to a study done by Du et al. [3], in 91.7% of COVID-19 patients, the virus is detectable in the saliva, which suggests that the active virus in the patient’s saliva can be spread by coughing and sneezing. Liu et al. [4] pointed out that COVID-19 can also infect patients through the eyes in addition to the respiratory tract. He pointed out that Guangfa Wang, a China Pneumonia Expert Group member, reported his infection with COVID-19 during the investigation stay in Wuhan. Even though a N95 mask was worn at all times, Wang’s eyes were exposed without protection. Two days before the onset of COVID-19, Wang had the phenomenon of red eyes. Hence, it is inferred that COVID-19 can infect humans when the eyes are exposed to the virus. Tang et al. [5] categorized the previous studies on aerosol transport and proposed a method for reducing infection caused by aerosol spreading. The literature review discussed the importance of controlling airflow direction to prevent the medical staff from inhaling virus-polluted air. Daniel et al. [6] studied the infection mitigation strategies in an operating room. Surgical procedures that generate aerosols (such as tracheal intubation, respirators, thoracotomy) were discussed. Aerosols were measured to evaluate each procedure with respect to the degree of particle generation. Chavez et al. [7] proposed the latest understanding and overview of COVID-19 for emergency medical staff, as well as management recommendations toward suspected patients. The article mentioned that COVID-19 mainly spreads through close contact (less than 6 ft) between people; therefore, there should be a 6-ft distance between each hospital bed; moreover, all the patients should wear masks, and the medical staff should wear suitable personal protective equipment.

To simulate the inhalation/exhalation of a human body, a simple version of a respirator with polyoxymethylene (POM) gear was fabricated using a 3D printer, in accordance with the design of Al Husseini et al. [8]. Lin et al [9]. experimentally investigated the flow characteristics and velocity fields in a typical operating room (with a laminar airflow ventilation system), to demonstrate the importance of the application of the ceiling return to entrainment of the contaminated air. This present experimental study will focus on the effect of different oxygen supply equipment on the droplets exhaled and dispersed by patients during intubation treatment. The impact of different oxygen supply equipment on the medical staff will be further studied experimentally by analyzing the variations of concentrations of PAO aerosols and the flow fields, which were measured by particle image velocimetry (PIV). Note that the PAO aerosols were released at the mouth of the manikin to simulate the aerosols produced during intubation processes.

## Methodology and equipment

### Laser sheet generator

**Fig. 1:**
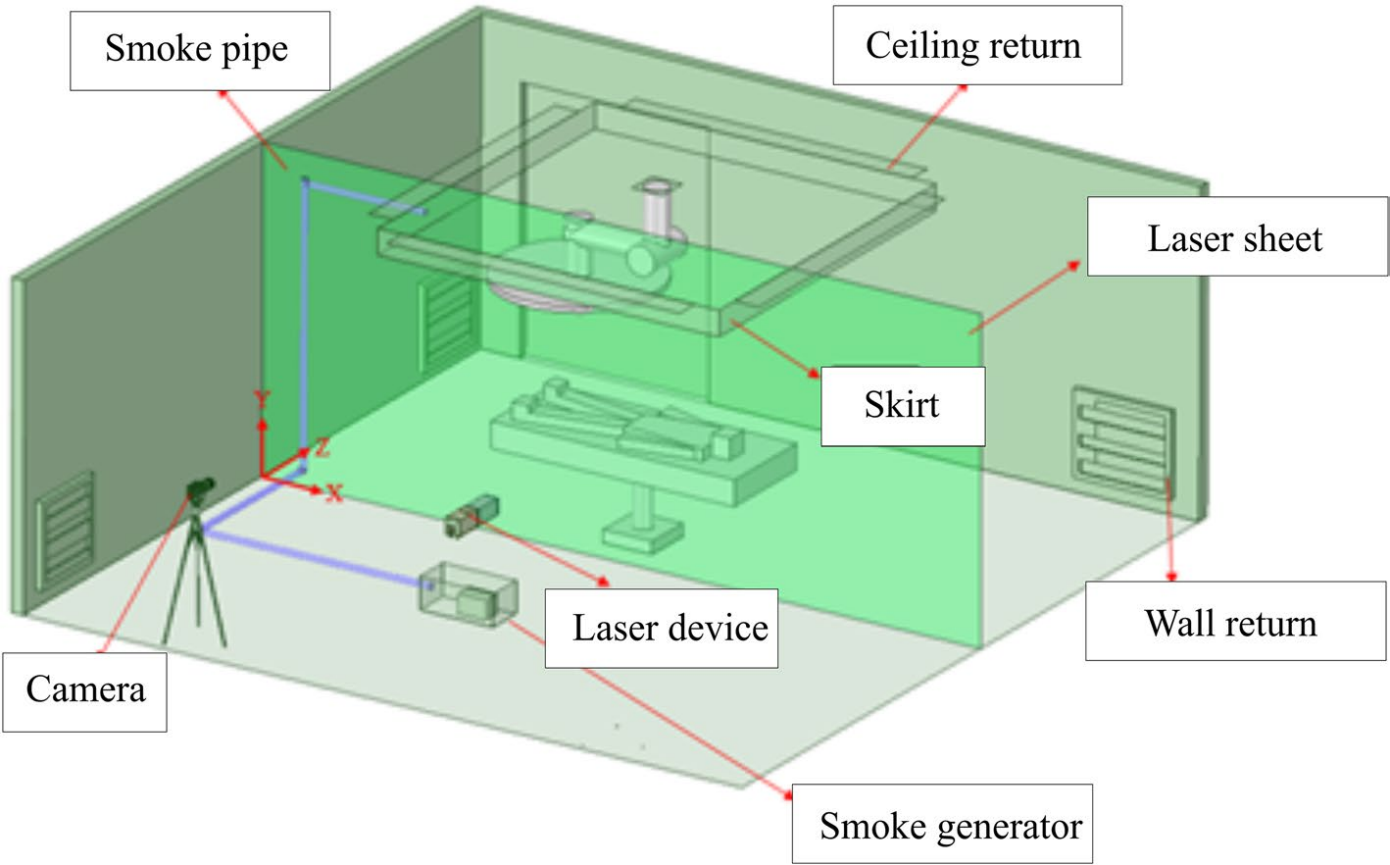
Experimental setup

The experimental setup is shown in Fig. 1, including a laser device, a high-speed camera, and a smoke generator similar to a previous study [9]. A laser sheet is first created in front of the high-speed camera in an operating room by the laser device, and then the smoke generator begins to generate the smoke. The laser device is based on the mechanism of a rotational mirror similar to the method that was proposed by Brucker et al. [10]. A homemade smoke generator was designed to generate a high-quality and uniform smoke flow pattern in the area of the flow visualization. The laser device and smoke generator that are used in this study are upgraded versions of those applied in a previous study [9]. When the smoke flows to the laser sheet, the reflected images are captured by the high-speed camera. Finally, the smoke movement images are analyzed by PIV to further characterize the indoor flow field. The most critical factor influencing the flow field visualization is the light source; in this experiment, a 2-W diode-pumped solid-state laser (DPSSL) is in use, which provides a compact light with a wavelength of 532 nm.

**Fig. 2:**
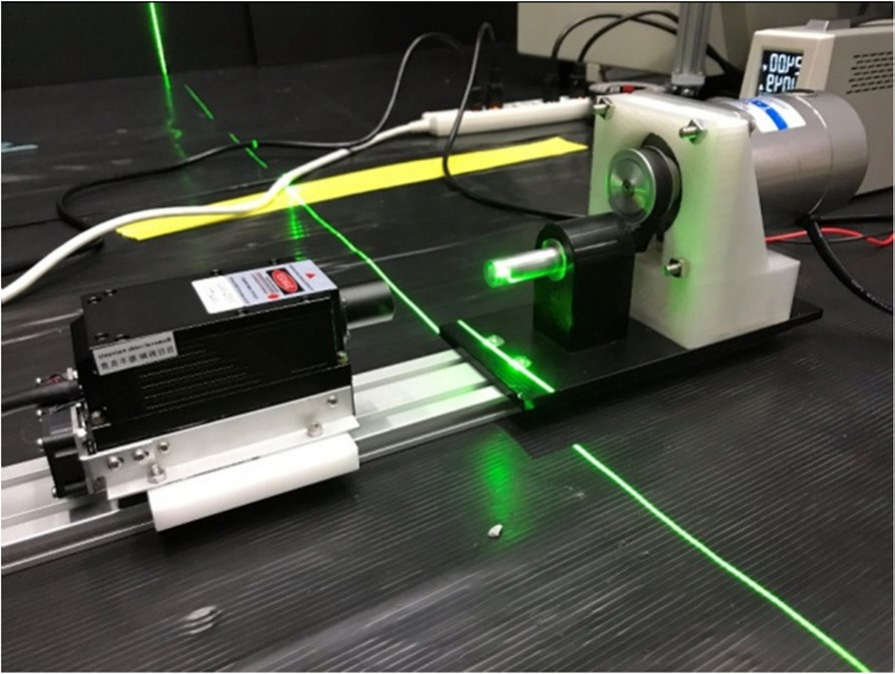
Laser sheet generator

To account for the fact that the intensity of the laser light will weaken with increasing irradiation distance, the laser imaging scanning system developed by our laboratory is used in this experiment, as shown in Fig. 2. Its major advantage is that the intensity of the laser light will not decay over time, and it can measure the airflow over an extensive area.

### Aerosol source

The type of aerosol chosen for this study was based on the US and EU standards for HEPA/ULPA filters. Those aerosols are DOP, DEHS, PAO, and PSL. In the beginning, the US Department of Defense used DOP to test the air filter filtration efficiency. Vapor DOP is irritating to the eyes and respiratory system of humans leading to this being replaced by PAO, which is reported to be safe, not corrosive, stable, and low-cost. PAO is now widely used to test air filter efficiency; however, after the test, the remaining liquid on the filter acts the same as DOP, which can only be gradually released and cause pollution of the test environment.

**Fig. 3:**
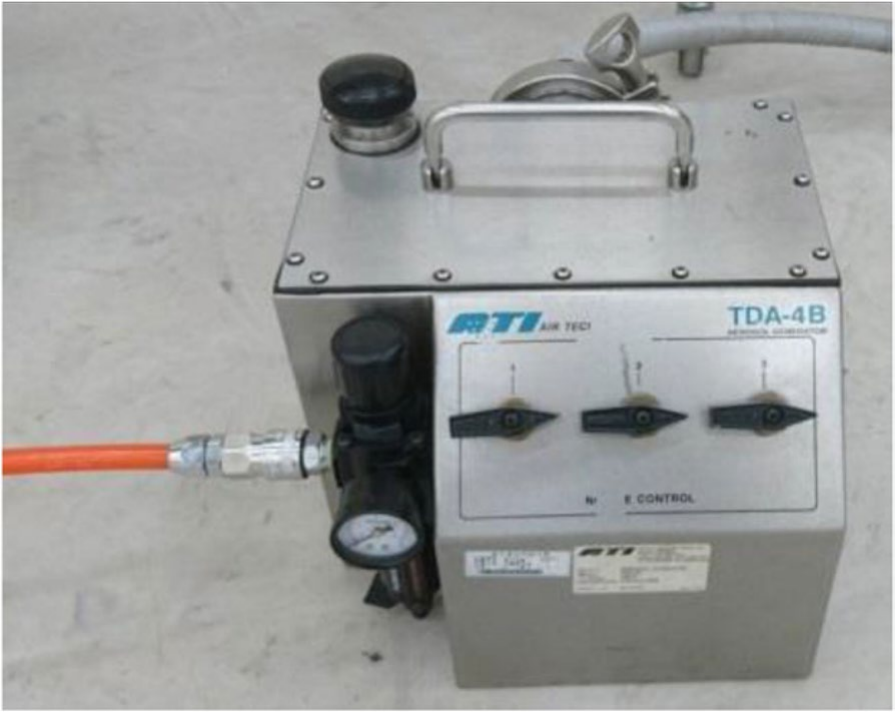
Aerosol generator

**Fig. 4:**
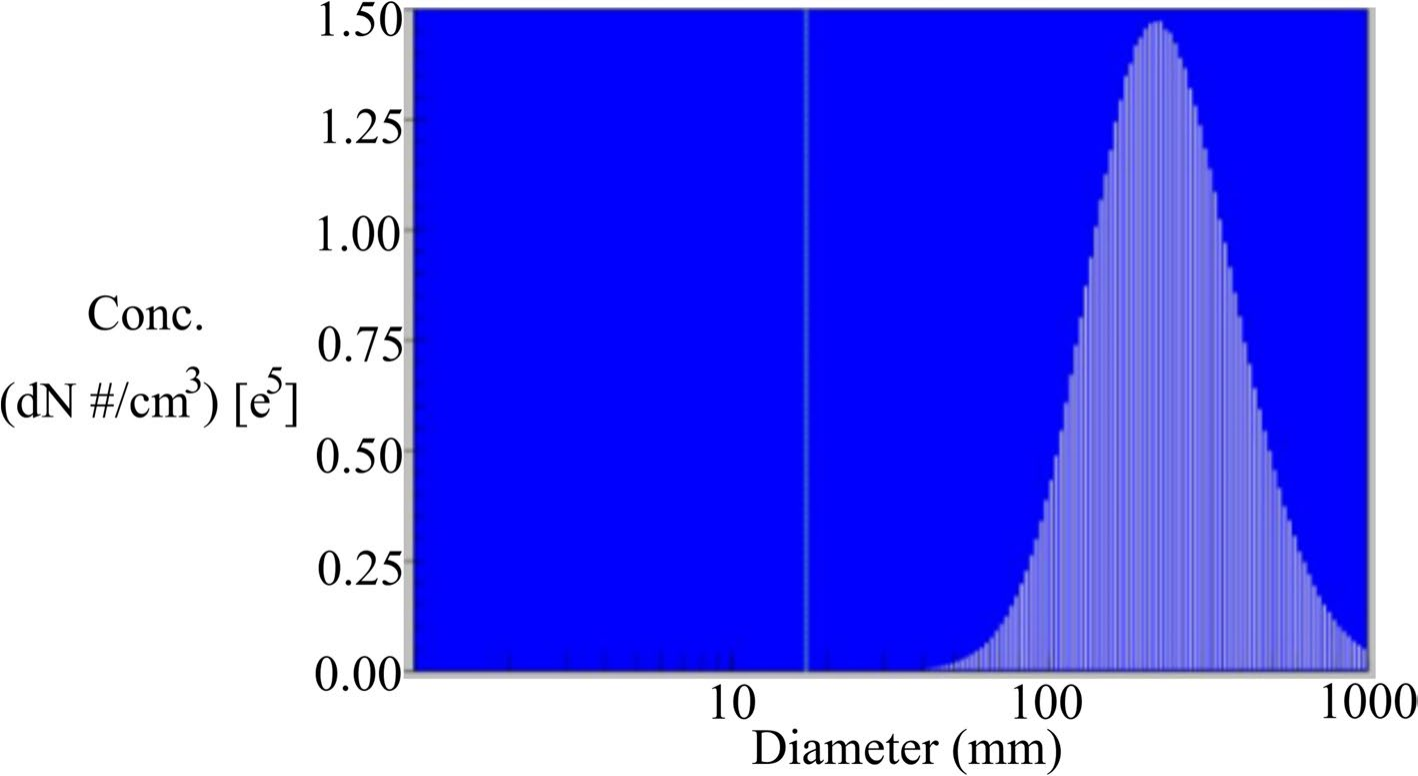
Particle diameter from the aerosol generator

In this experiment, the oily aerosol generator (model type: ATI TDA-4B), as shown in Fig. 3, is adopted to generate PAO for use as testing particles. Similar to the particles caused by patients’ vomiting during intubation, most of the generated particles have a diameter of approximately 0.5 μm, as shown in Fig. 4; generator type is 1 to 6 Laskin nozzle, the released concentration is about 100 μg/l, flowrate 810 CFM (22,923 l/min), the compressed gas has the minimum level at 2.65 CFM (74.995 l/min)/nozzle @ 20/23 psig. This device is manufactured with the industry standards Laskin III-A that is suited to the requirements of this study.

### Aerosol photometer

**Fig. 5:**
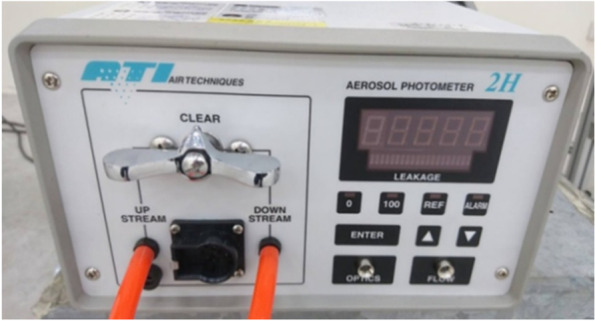
Aerosol photometer

The aerosol generator shown in Fig. 3 operates in conjunction with an aerosol photometer to measure aerosol penetration during the experiments. The aerosol photometer used in this study (model type: ATI 2H), as shown in Fig. 5, takes the variable concentration sample of PAO aerosol from various monitoring points close to the patient to compare the effect of the patient’s exhaled smoke upon the medical staff. The tested concentration range is between 0.00005 and 120 μg/l, with a sampling error of ± 1%, and the sampling frequency is 1 CFM (28.3 l/min), as defined by the device specifications. The accuracy and repeatability for the amplifier used are 1% and 0.5% of the full scale, respectively, which are suitable for this study.

### High-sensitivity camera

**Fig. 6:**
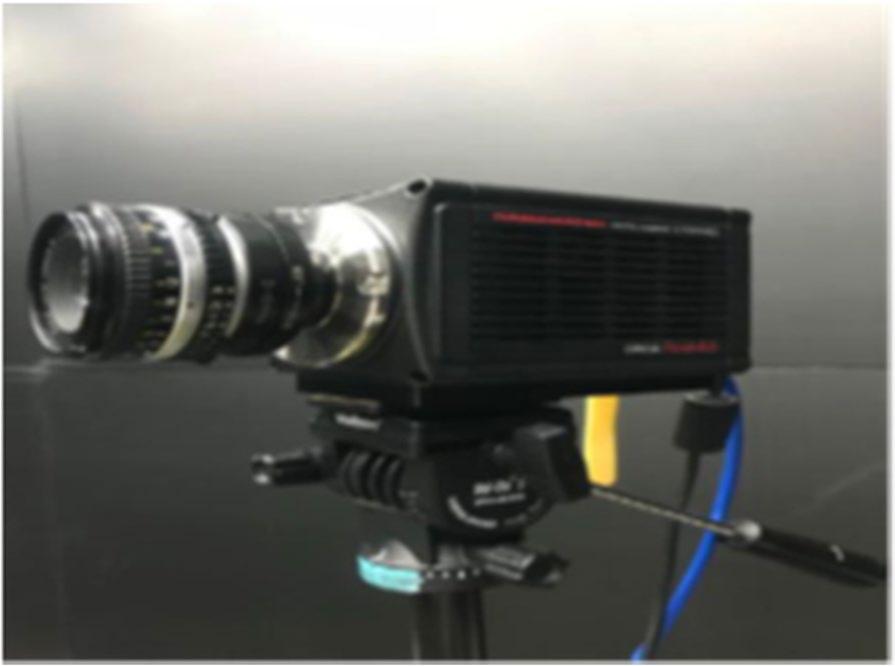
High-sensitivity camera

The high-sensitivity camera (model type: HAMAMATSU, ORCA-Flash 4.0C13440-20fCU), as shown in Fig. 6, is used to record different flow field conditions as per the previous study [9]. Parameters such as exposure time, sensitivity, frame number, and pixels can be adjusted by connecting the camera to the computer through USB3.0 and using the integrated software interface. The camera is equipped with a scientific complementary metal-oxide semiconductor (sCMOS) imaging sensor; in addition, it has a 2048 × 2048 (V) resolution and a maximum 100 fps imaging speed. The image frequency and exposure time were set to 60 fps and 14 ms, respectively, during the experiments. This provides high-quality smoke flow pattern images and PIV results.

### Resuscitation ball

**Fig. 7:**
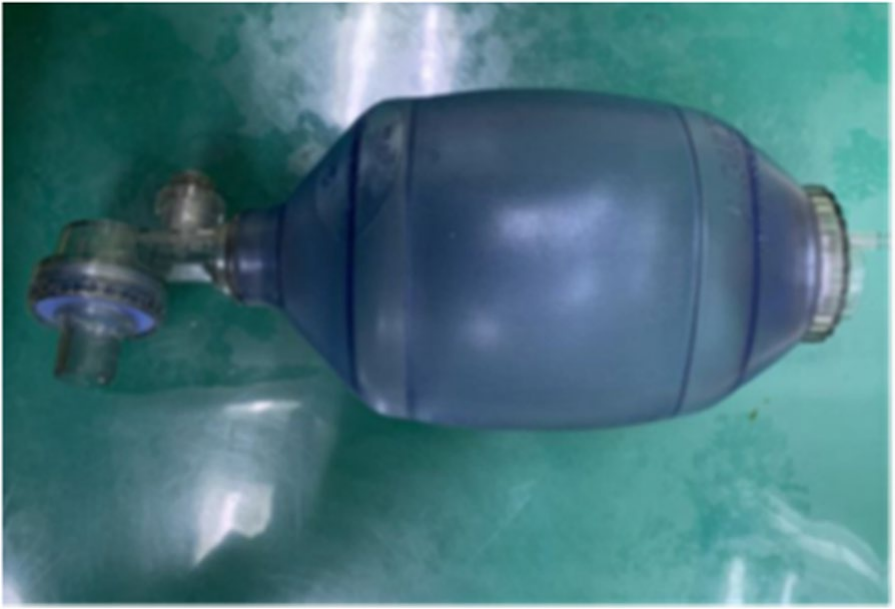
Resuscitation ball

To reproduce the action of human lungs breathing, the resuscitation ball with a capacity of 1000 ml is used, as shown in Fig. 7. Exhale when the resuscitation ball is squeezed and inhale when it is relaxed.

### Simple respirator

**Fig. 8:**
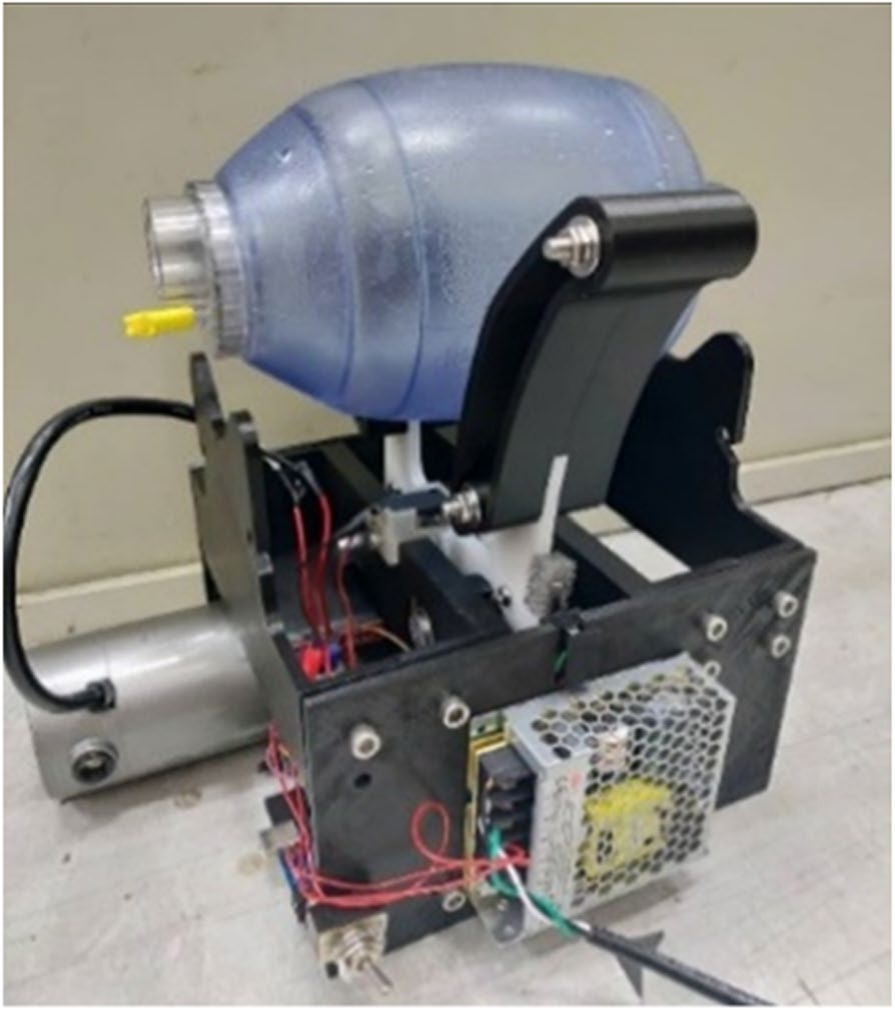
Respirator

**Fig. 9:**
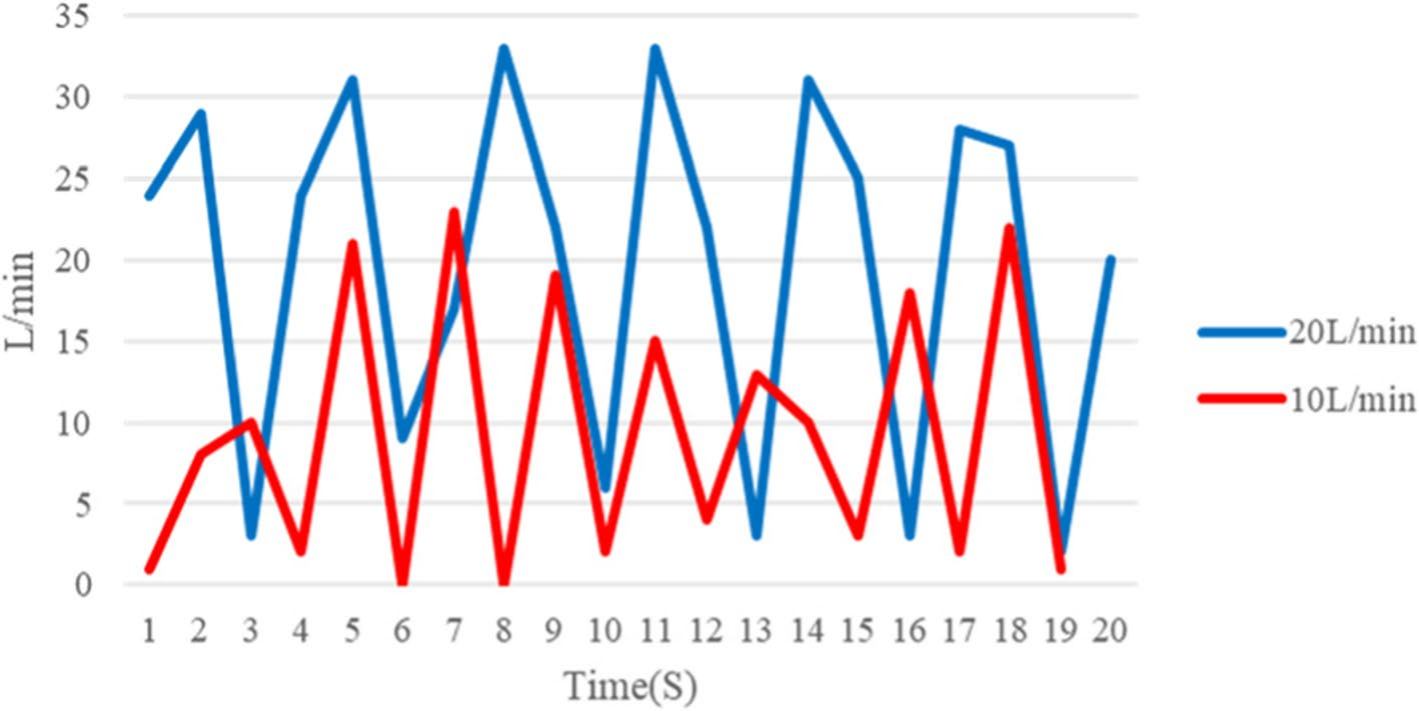
Tidal volume variation of the respirator

An automatic squeezing device is made to stabilize the tidal volume of the respirator model. This device references and simplifies the MIT Emergency Ventilator model, while a 3D printer fabricated the structure of the entire device; the gear is made of polyoxymethylene (POM) plastic steel to enhance the mechanical strength. The complete respirator is shown in Fig. 8. Using a 24-V DC motor, along with Arduino and a motor driver (L298n) to control the motor rotation parameter, the rate of breath and the tidal volume can be stabilized. Figure 9 shows that the simple respirator model performs 30 breaths per minute. The variation of tidal volume is 35 times per minute.

### Medical dummy

**Fig. 10:**
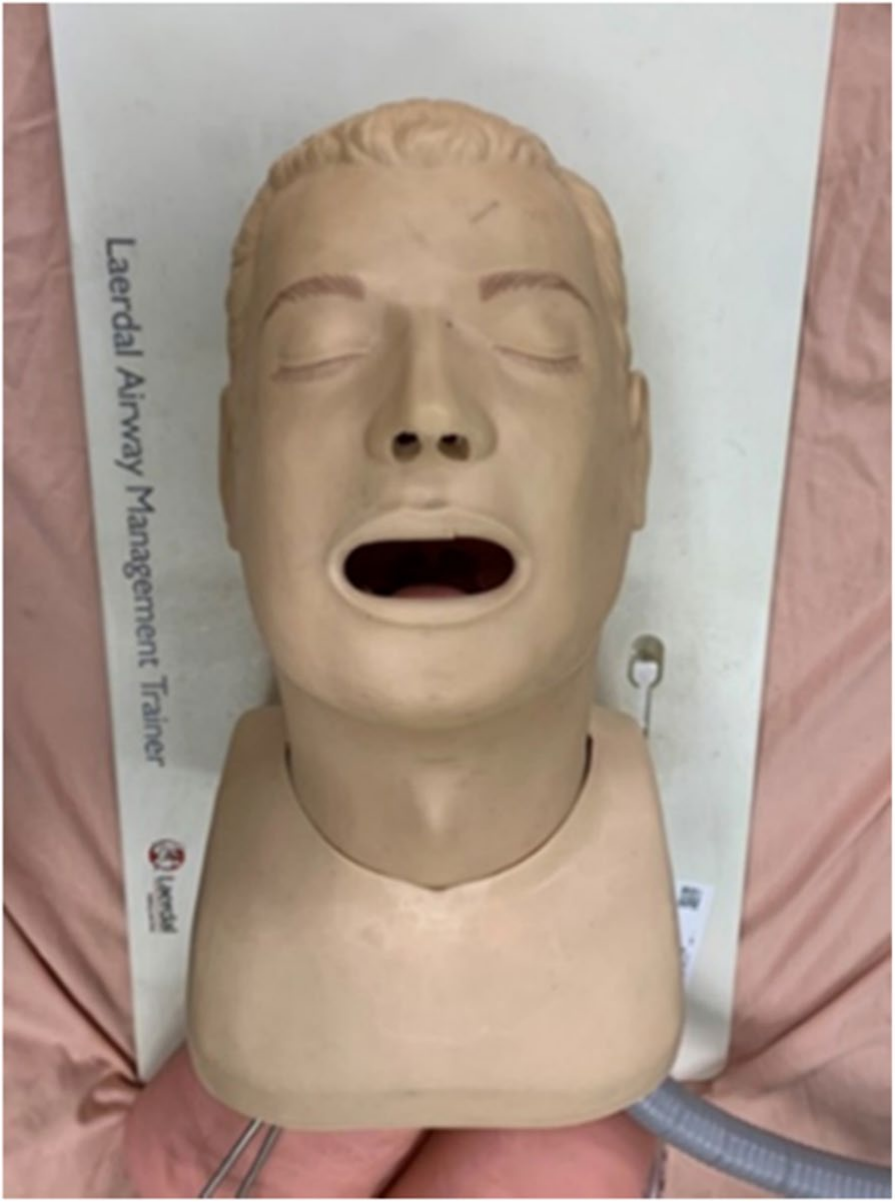
Medical dummy

This experiment uses a medical dummy, which has three organs: esophagus, trachea, and lung. Both PAO aerosol and flow field visualization smoke are exhaled from the dummy. The dummy size is 45 cm × 25cm × 25 cm, as shown in Fig. 10.

### Nasal cannula (HFNC)

**Fig. 11:**
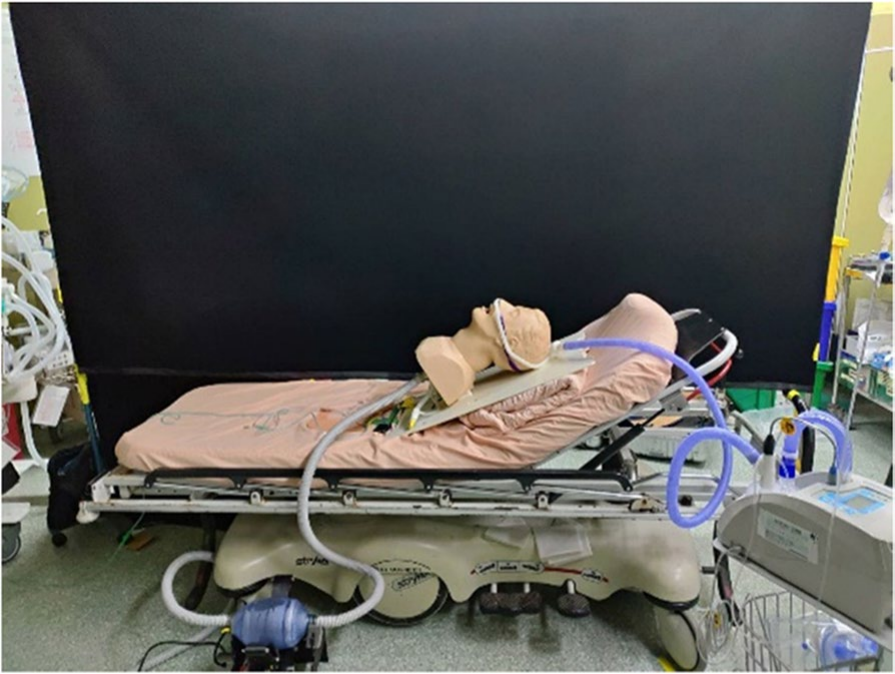
High-flow nasal cannula (HFNC)

**Fig. 12:**
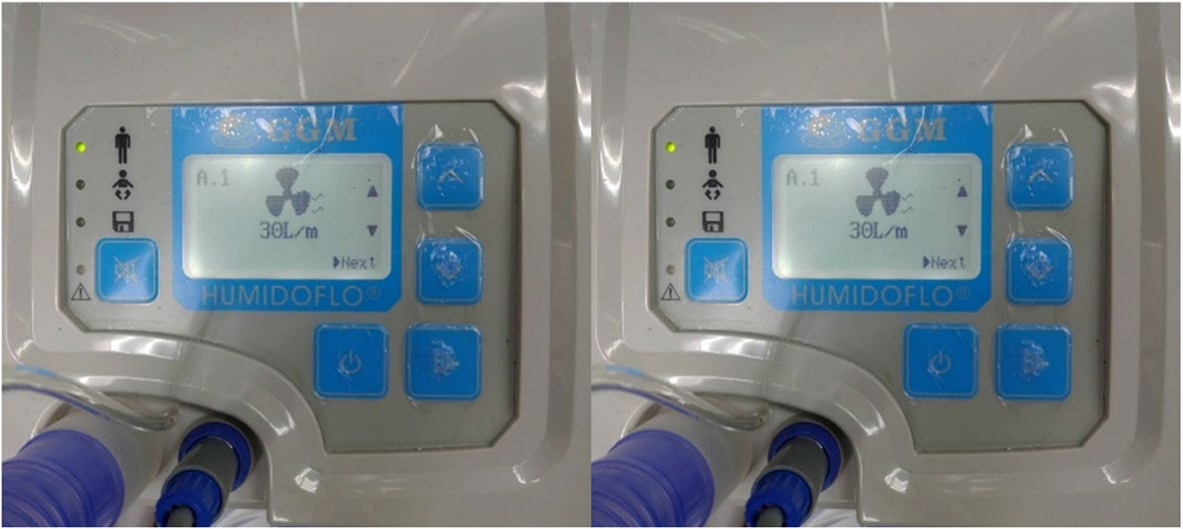
Nasal cannula

**Fig. 13:**
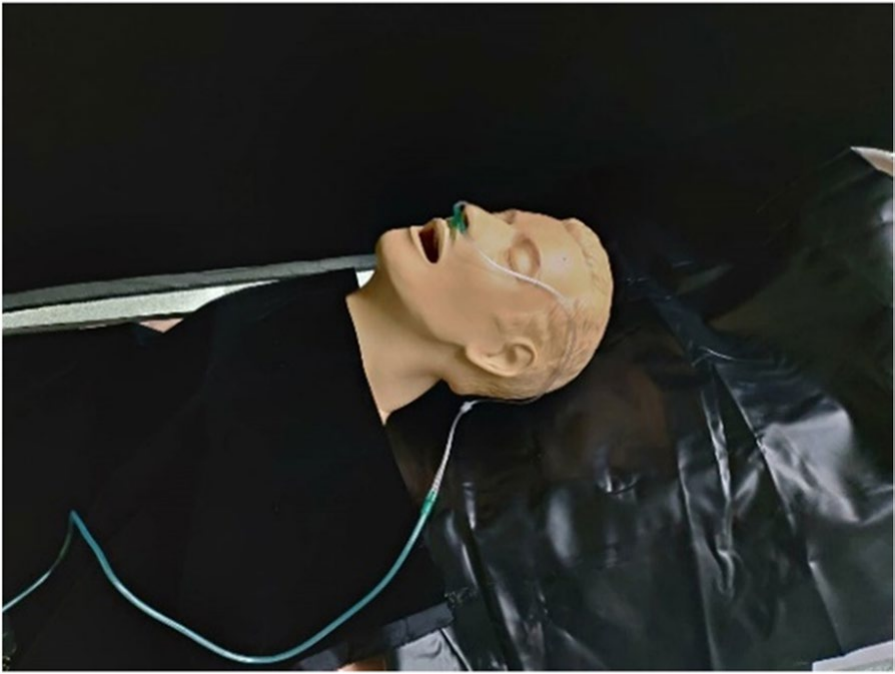
Thirty liters per minute and 70 l/min

In this experiment, two types of nasal cannulas are in use: high-flow nasal cannula (HFNC) and nasal cannula (N/C), as shown in Figs. 11 and 12, respectively. HFNC supplies oxygen at two flow rates, 30 l/min and 70 l/min, as shown in Fig. 13; N/C supplies oxygen at 15 l/min.

### Non-rebreathing mask (NRM), continuous positive airway pressure (CPAP), and ventilator-assisted pre-oxygenation (VAPOX)

**Fig. 14:**
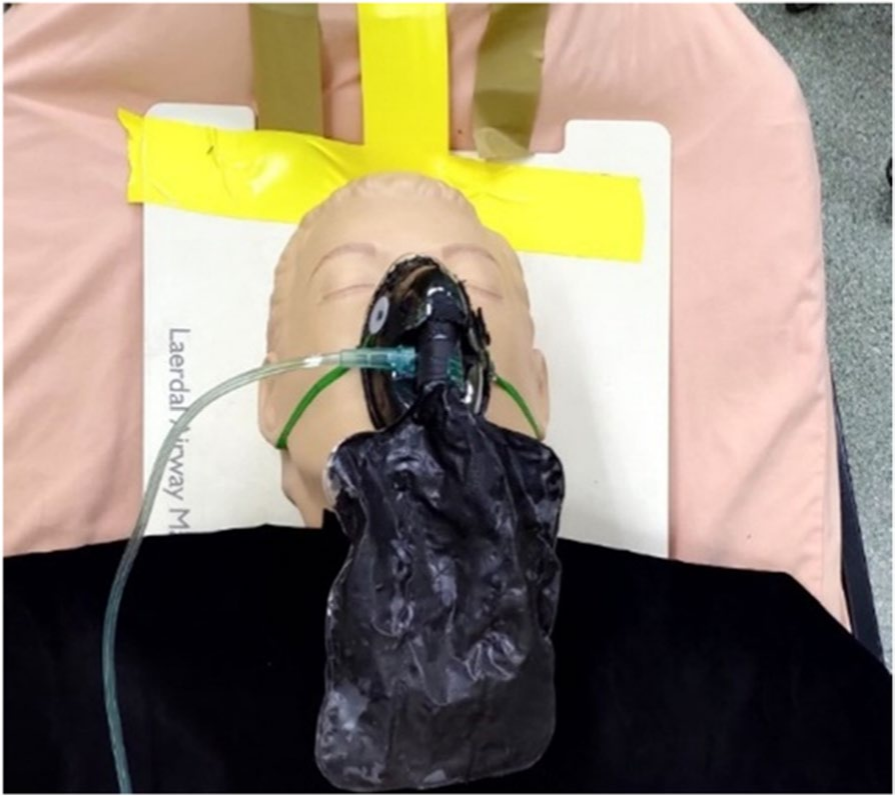
Non-rebreathing mask (NRM)

The non-rebreathing mask (NRM), Fig. 14, directly supplies oxygen to the patient’s nose and mouth to increase the inhaled oxygen concentration. NRM is similar to an oxygen mask, but with an additional airbag and a one-way valve, which allows the carbon dioxide exhaled by the patient to be discharged out of the mask. The supplied oxygen flow rate of the NRM is 15 l/min.

**Fig. 15:**
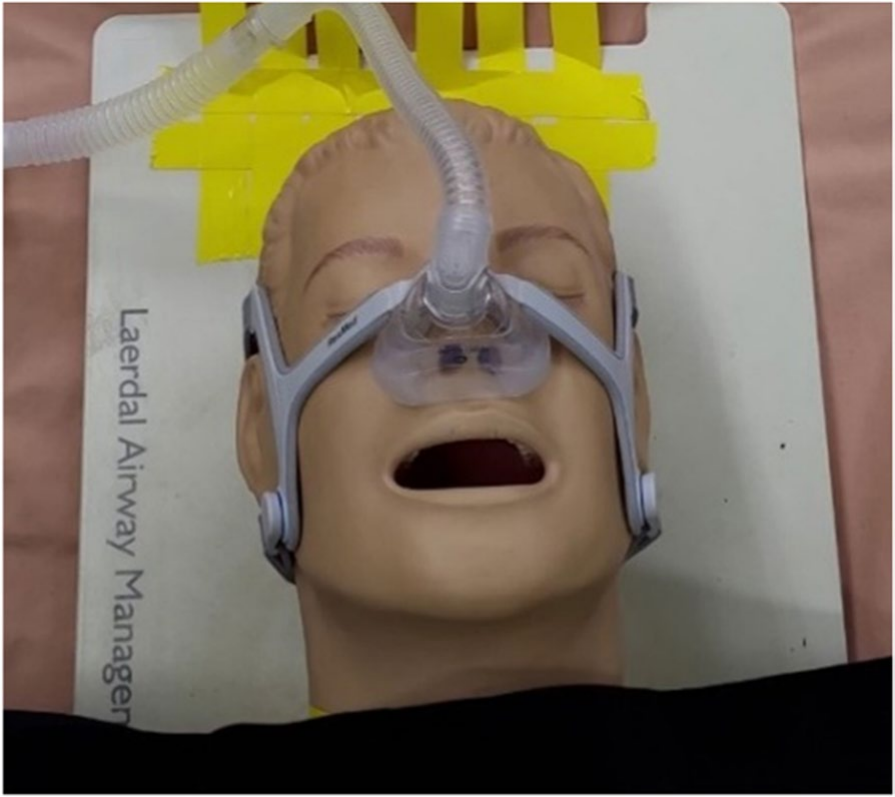
Continuous positive airway pressure (CPAP)

**Fig. 16:**
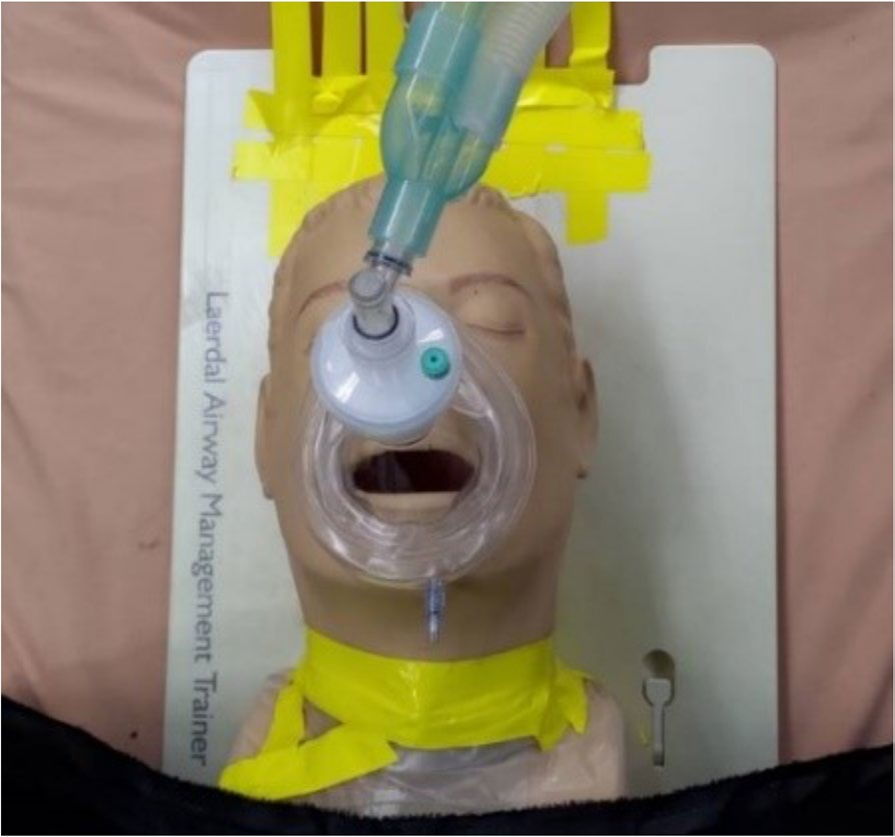
Ventilator-assisted pre-oxygenation (VAPOX)

For CPAP and VAPOX, as shown in Figs. 15 and 16, respectively, the supplied oxygen flow rates are 40 l/min and 28 l/min, respectively. Capecitabine and oxaliplatin (CAPOX) is a chemotherapy drug prescribed to provide oxygen to the patient [11]. The primary advantage of using VAPOX is its good airtightness, which makes any potentially infective agents in the patient’s exhaled breath unable to diffuse to the surrounding environment.

### Experimental environment

**Fig. 17:**
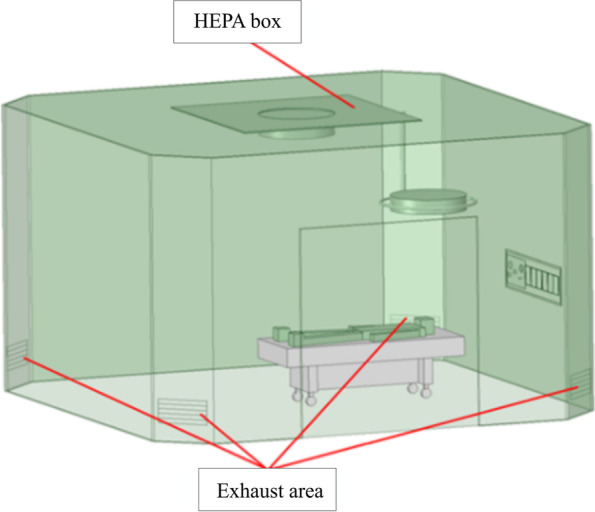
Schematic diagram of the emergency room

The experiment takes place in an emergency room of Taipei City Hospital Zhongxing Branch. A 3 m × 3 m filter chamber (HEPABOX) is installed on the ceiling, supplying an airflow rate of 0.119 m/s. Four return air inlets are located at floor level within the emergency room, one at each corner. The schematic diagram of the emergency room is shown in Fig. 17.

### Experiment method

#### PAO aerosol concentration measurement

**Fig. 18:**
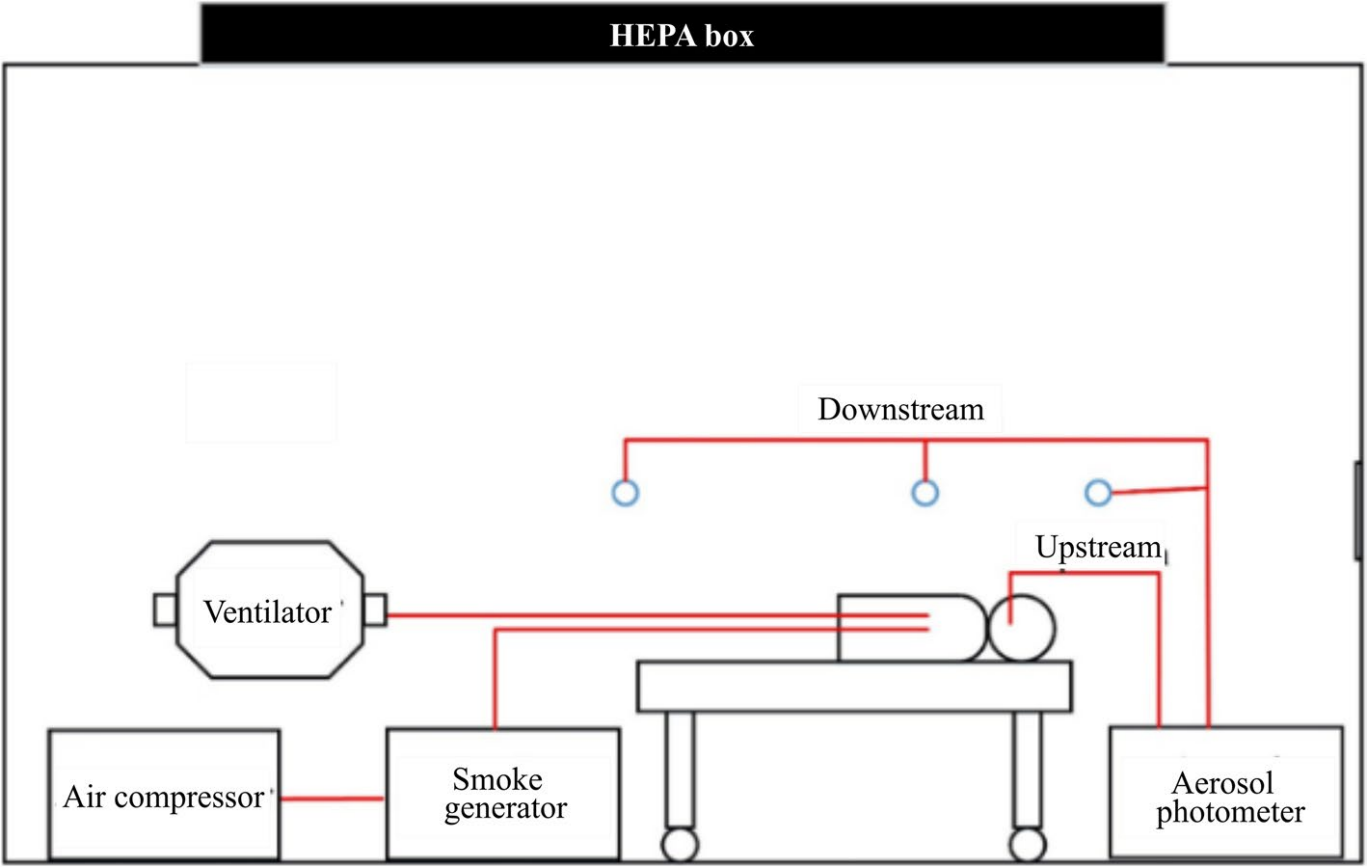
Experiment system

**Fig. 19:**
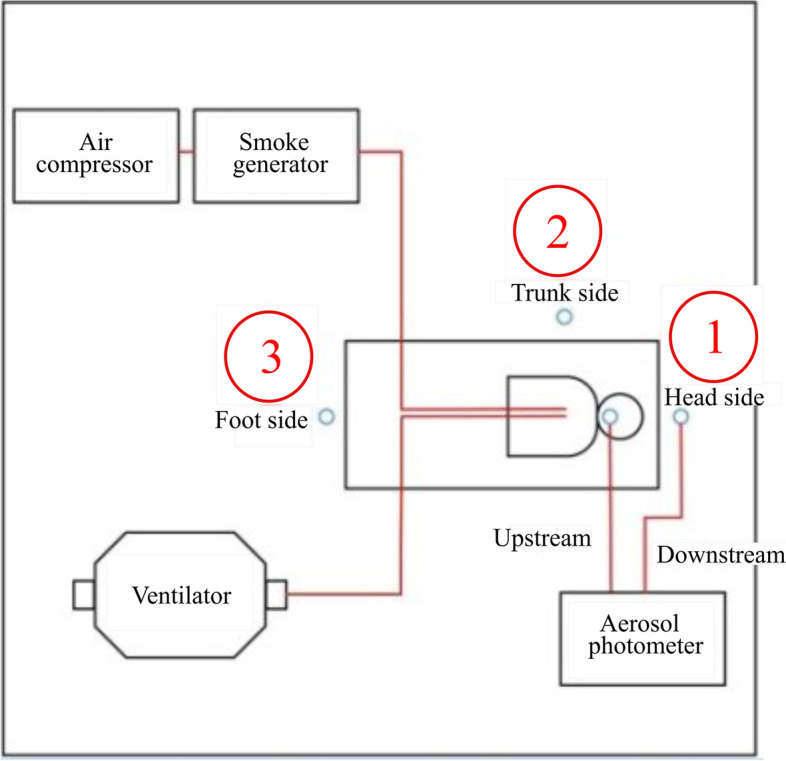
Measurement spots

Determination of aerosol diffusion is applied to verify and quantify the impact of a patients’ rapid exhalation on the medical staff. An oil-free air compressor is used to start the smoke generator, test particles of PAO are released, then the aerosol photometer is used to measure the pollutant concentration upstream and downstream of the smoke source. Downstream of the smoke source includes the head side, trunk side, and foot side. The height of the measurement point is 20 cm above the dummy’s mouth. The variation of the PAO concentration at each point is recorded for further analysis and comparison. The schematic diagram of the experiment system is shown in Fig. 18; the measurement point is shown in Fig. 19.

#### Using PIV to do flow field visualization

**Fig. 20:**
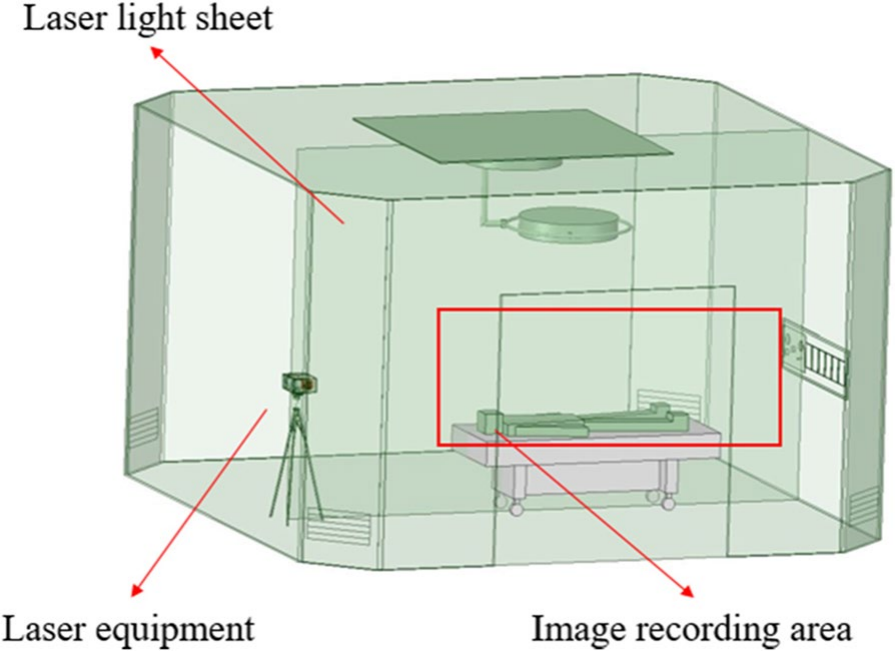
Laser sheet on the front face

**Fig. 21:**
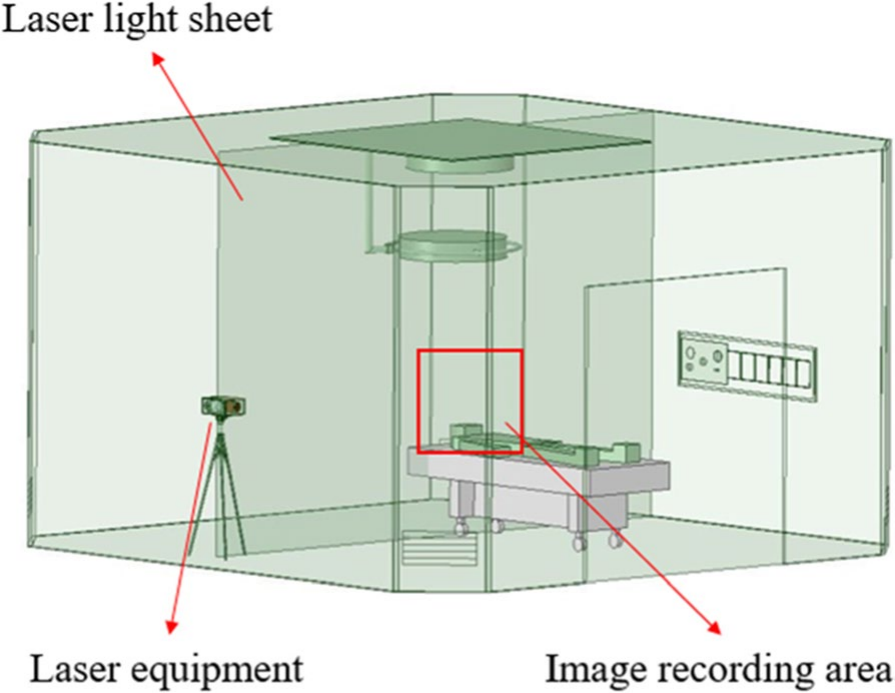
Laser sheet at the side face

**Fig. 22:**
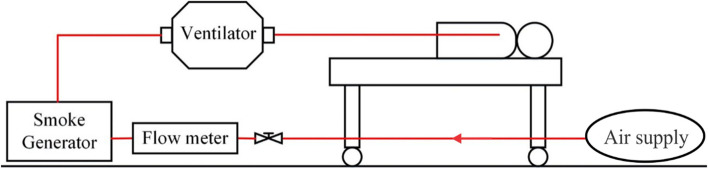
Flow chart of the smoke generation process

In this experiment, the area around the head is the main observation area. Hence, two laser sheets are created, one at each side of the dummy’s head as shown in Figs. 20 and 21. The high-speed camera is installed in front of the laser sheet, and the smoke generator is placed in a storage box and controlled by a remote controller. A needle valve and a flow rate meter are used to control the amount of compressed air entering the storage box. With the assistance of the respirator and resuscitation ball, the smoke can be consistently generated. The smoke generation process is shown in Fig. 22.

#### Test condition

In this experiment, six different oxygen supply systems within two different tidal volumes are tested, as shown in Table 1. This is to test the impact of aerosol diffusion under different oxygen supply rates and tidal volumes. Tidal volume at 10 l/min occurs when an adult is resting; tidal volume at 20 l/min occurs when an adult has rapid breathing. Of the six different oxygen supply systems, HFNC, N/C, and NRM supply the oxygen consistently, while CPAP and VAPOX supply intermittently.

**Table 1:**
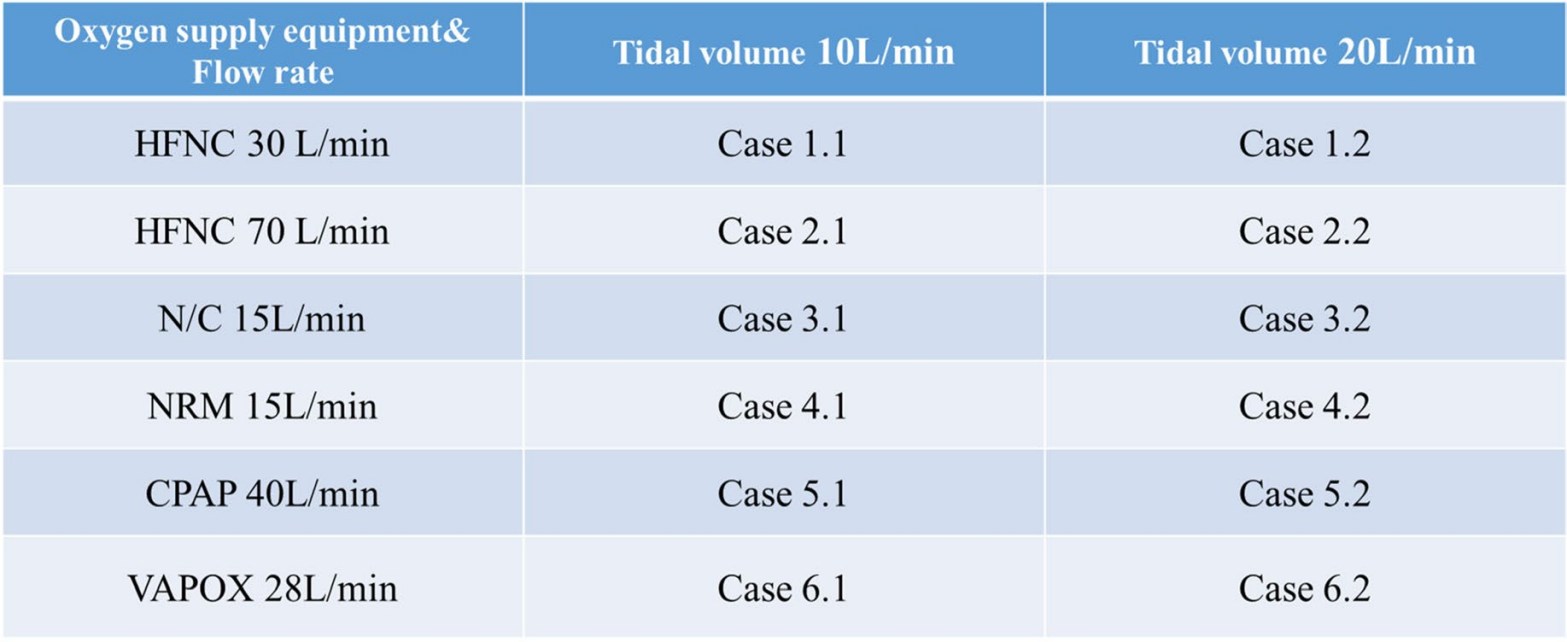
Test condition

## Result and discussion

### Flow field visualization analysis

#### Emergency room flow field

**Fig. 23:**
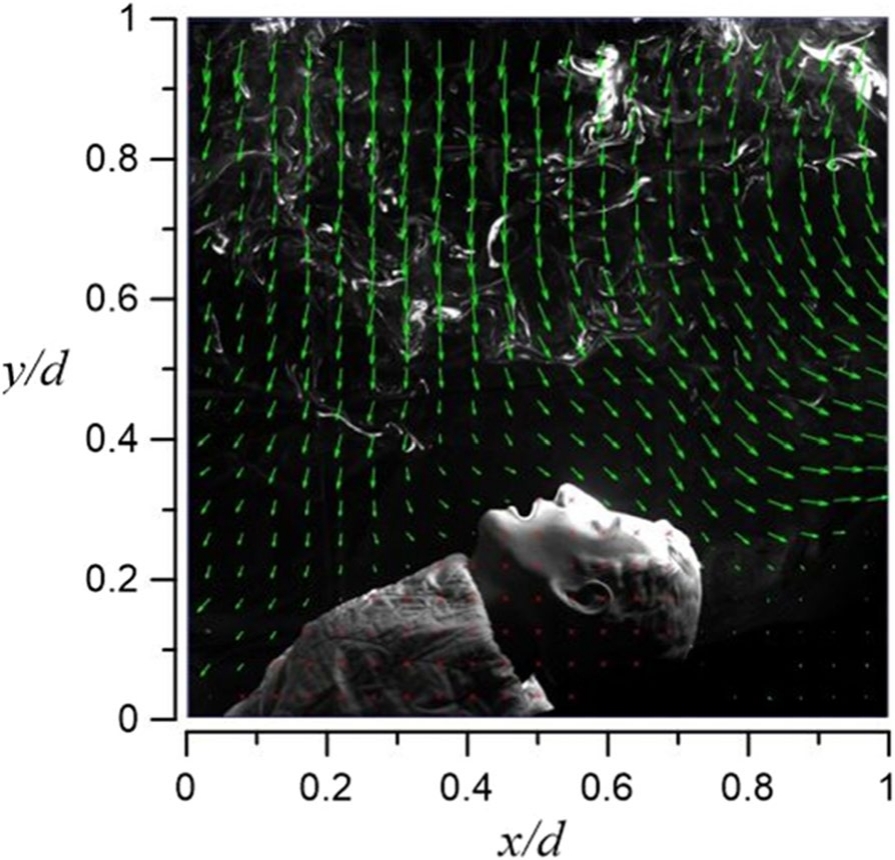
The side view flow visualization for the mean flow field, *d* = 120 cm

**Fig. 24:**
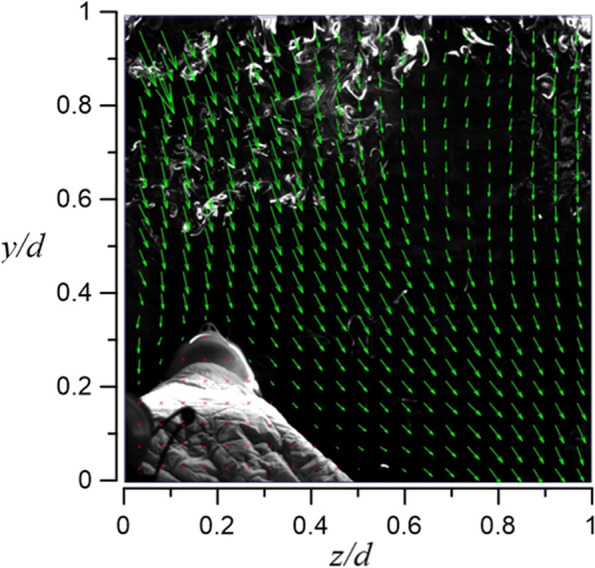
The face view flow visualization for the mean flow field, *d* = 106 cm

Figures 23 and 24 demonstrate the visualization of the mean flow field in the emergency room. *x*, *y*, and *z* are the horizontal, vertical, and axial coordinates, respectively (as shown in Fig. 1 with red color vectors). *d* is the real size of the PIV-measured area in the corresponding direction. This technique is used to nondimentionalize the horizontal, vertical, and axial distances homogeneously. The velocity field is observed and processed based on the algorithm that was proposed by Thielicke and Stamhuis [12]. The uncertainty of the PIV results in this study is estimated to be ± 2% [13]. In Fig. 22, it is clear that HEPA BOX influences the room airflow; hence, all airflows move downward. When the airflow comes into contact with the dummy and the hospital bed at an elevation angle of 30°, it spreads toward the head and feet. From Fig. 23, it is seen that the airflow from HEPA BOX spreads to the two sides after encountering the dummy and the hospital bed. Through the flow field visualization’s average flow field visualization, the airflow in the experimental site is observed to be stable, and there is no apparent turbulence and deflection. The camera’s shooting range of the front face is 120 cm × 120 cm, while the side face is 106 cm × 106 cm.

#### High-flow nasal cannula (HFNC)

The exhaled smoke flow field visualization result of using a high-flow nasal cannula (HFNC) on the dummy is demonstrated and discussed in this section. In total, four test conditions are implemented (case 1.1, case 1.2, case 2.1, case 2.2). The two variables are oxygen supply rate (30 l/min and 70 l/min) and dummy’s tidal volume (10 l/min and 20 l/min).

**Fig. 25:**
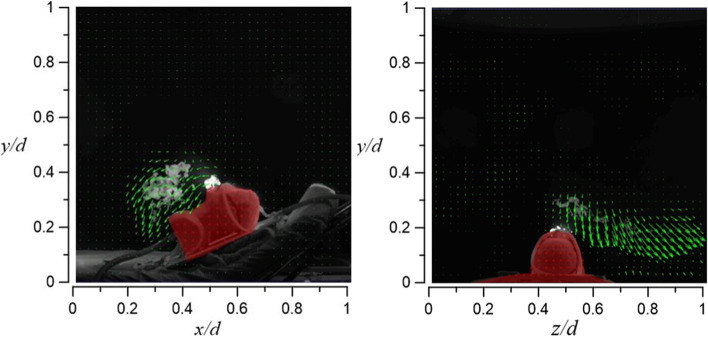
Flow field visualization result of case 1.1 (left: side view; right: face view)

Figure 25 shows the room’s flow field visualization result for case 1.1, in which HFNC supplies oxygen at a flow rate of 30 l/min and dummy’s tidal volume 10 l/min. The exhaled airflow rises about 16 cm and is unable to continue to rise due to the downward airflow from the HEPA BOX; the exhaled airflow then diffuses toward the return air inlet. Through Fig. 25, it can be observed that most of the smoke appears on the right side of the dummy (axial level of 0.5 < *z*/*d* < 1).

**Fig. 26:**
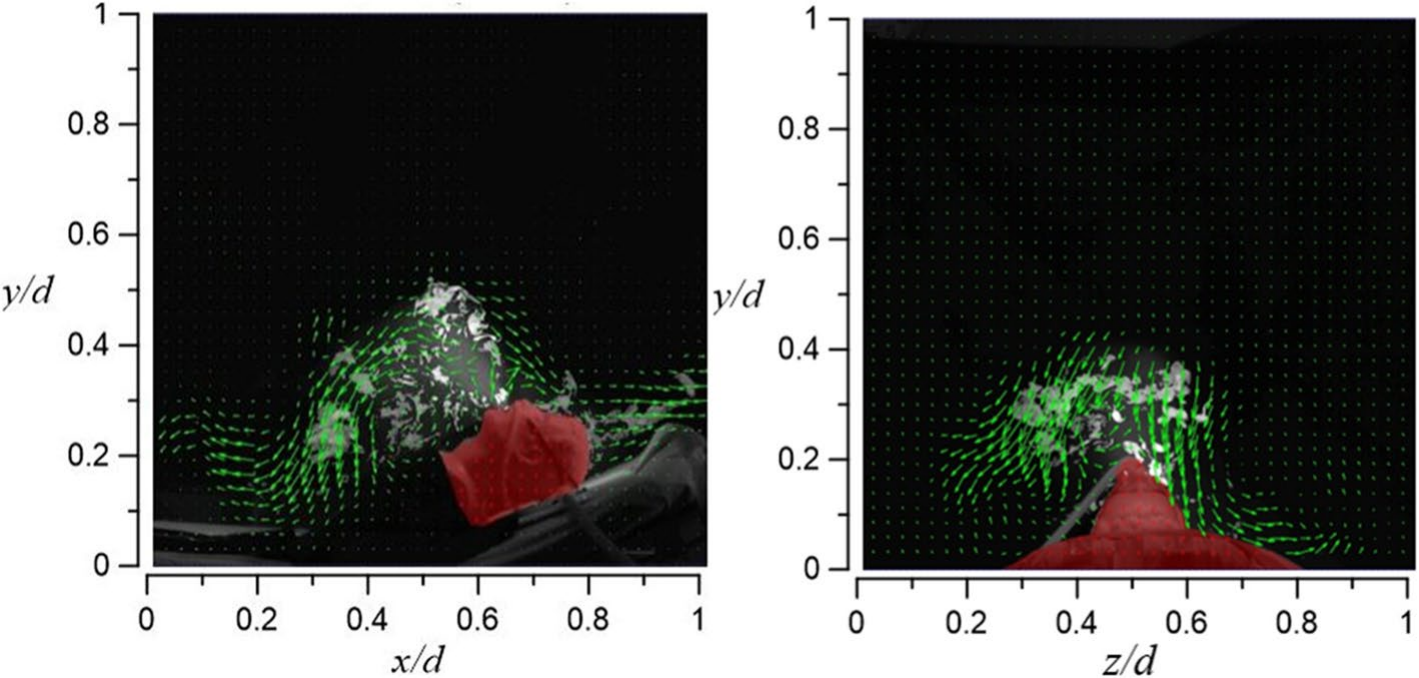
Flow field visualization result of case 1.2 (left: side view; right: face view)

Figure 26 shows the result of case 1.2 (HFNC oxygen supply rate 30 l/min; dummy’s tidal volume 20 l/min). Compared with case 1.1, the exhaled airflow rises higher to about 28 cm. This is because of the larger tested tidal volume. As observed in Fig. 26, smoke begins to appear on the dummy’s left side due to a larger amount of exhaled smoke (axial level of 0.2 < *z*/*d* < 0.5).

**Fig. 27:**
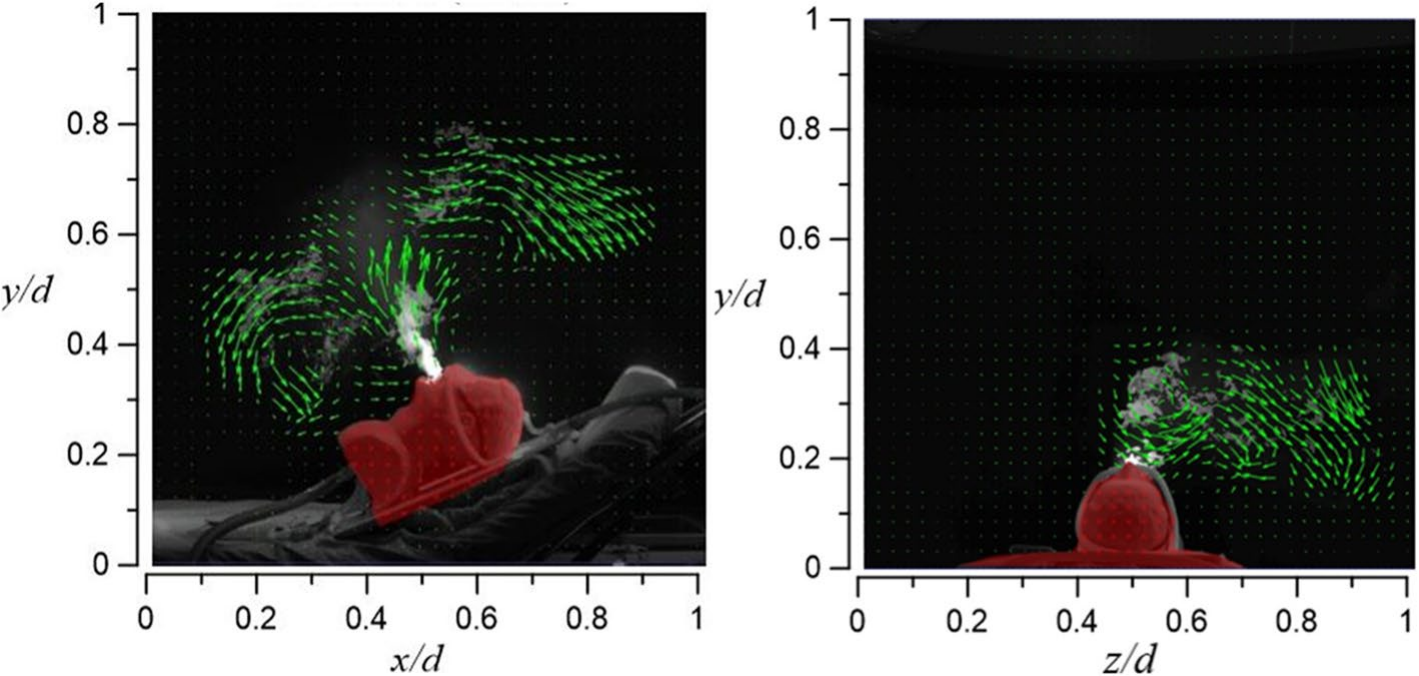
Flow field visualization result of case 2.1 (left: side view; right: face view)

In case 2.1 and case 2.2, HFNC has an oxygen supply rate of 70 l/min in both cases, and the tidal volume is 10 l/min and 20 l/min, respectively. Figure 27 shows the visualization result of case 2.1. By increasing the oxygen supply rate from 30 l/min (case 1.1) to 70 l/min, the smoke can rise to a height of 56 cm (vertical level of *y*/*d* ≈ 0.8), but the smoke still flows only to the right side of the dummy.

**Fig. 28:**
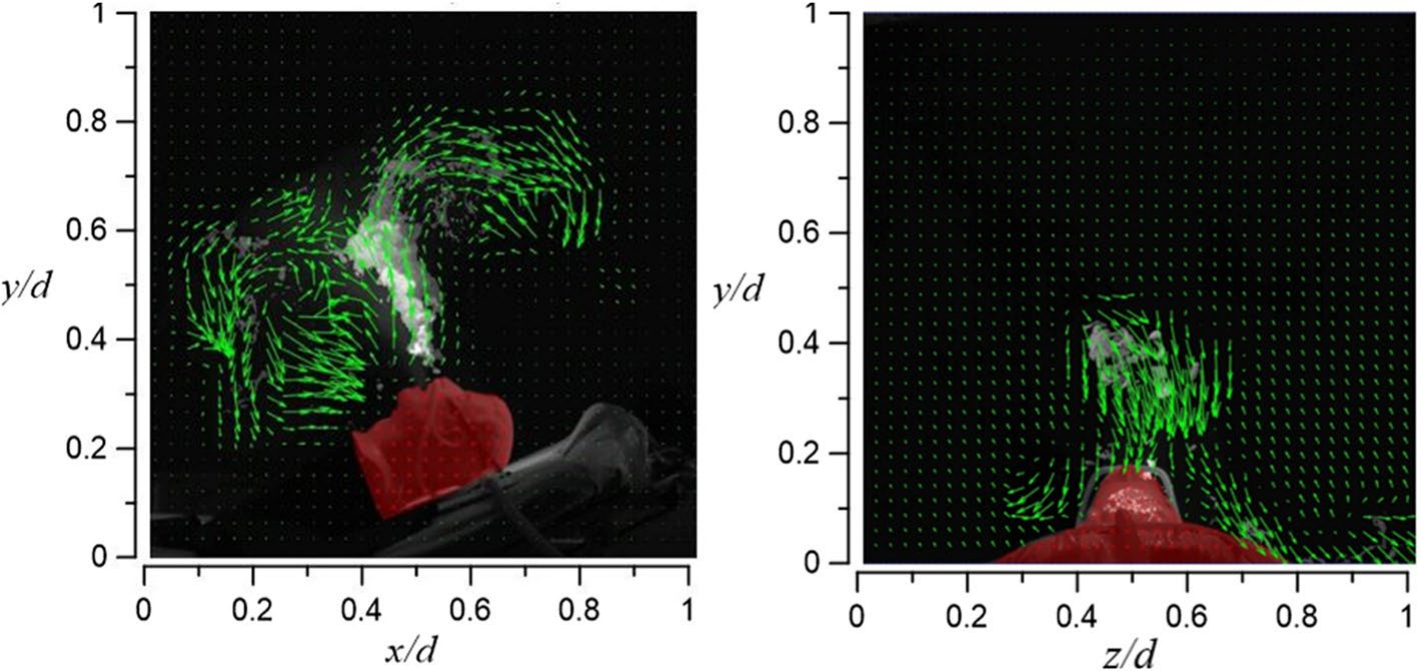
Flow field visualization result of case 2.2 (left: side view; right: face view)

Compared with case 2.1, case 2.2 raises the dummy’s tidal volume to 20 l/min, but the smoke only rises 5 cm higher (61 cm) (vertical level of *y*/*d* ≈ 0.85) compared to case 2.1. A possible explanation is that the closer the smoke gets to the HEPABOX, the encountered resistance increases. In Fig. 28, the smoke flows to both sides of the dummy (mostly at the axial level of 0.3 < *z*/*d* < 0.8) due to the larger amount of exhaled smoke, which also happens in case 1.2.

#### Nasal cannula (N/C)

Two test conditions are demonstrated in the nasal cannula experiment. The oxygen supply rate is fixed at 15 l/min; the only variable is the tidal volume (case 3.1, 10 l/min; case 3.2, 20 l/min).

**Fig. 29:**
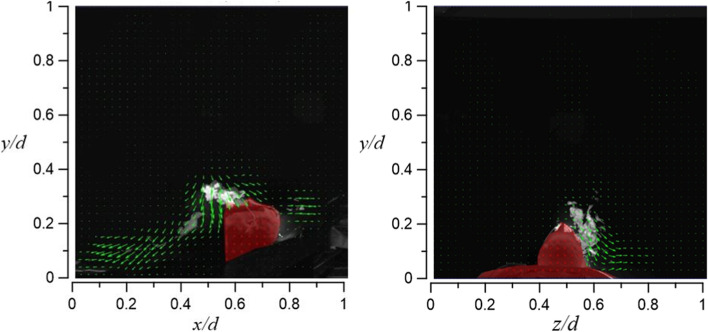
Flow field visualization result of case 3.1 (left: side view; right: face view)

**Fig. 30:**
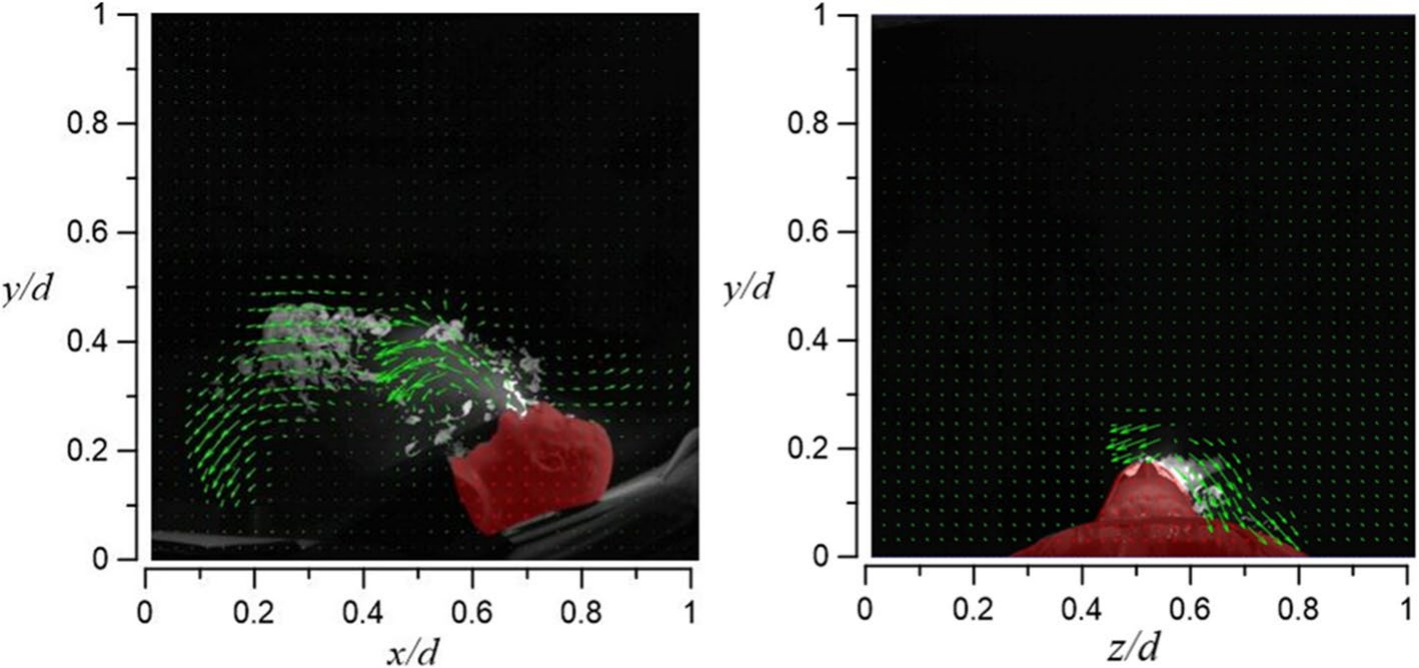
Flow field visualization result of case 3.2 (left: side view; right: face view)

Since the oxygen is supplied at a low rate (15 l/min), the smoke’s diffusion height is only about 12 cm (vertical level around *y*/*d* ≈ 0.35), and then, it is carried down by the airflow of the HEPABOX. With an increase in tidal volume from 10 to 20 l/min, the flow of the exhaled smoke changes from only appearing on the right side (axial level of 0.5 < *z*/*d* < 0,7) to appearing on both sides. The flow field visualization results of case 3.1 and case 3.2 are shown in Figs. 29 and 30, respectively.

#### Non-rebreathing mask (NRM)

**Fig. 31:**
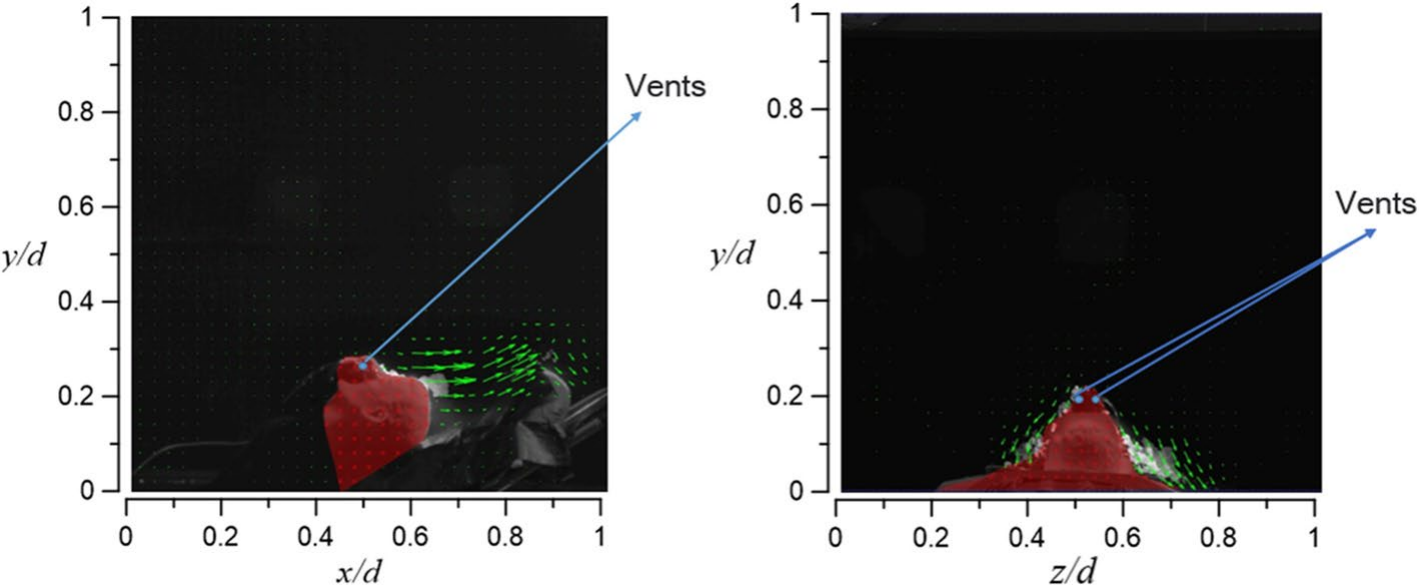
Flow field visualization result of case 4.1 (left: side view; right: face view)

**Fig. 32:**
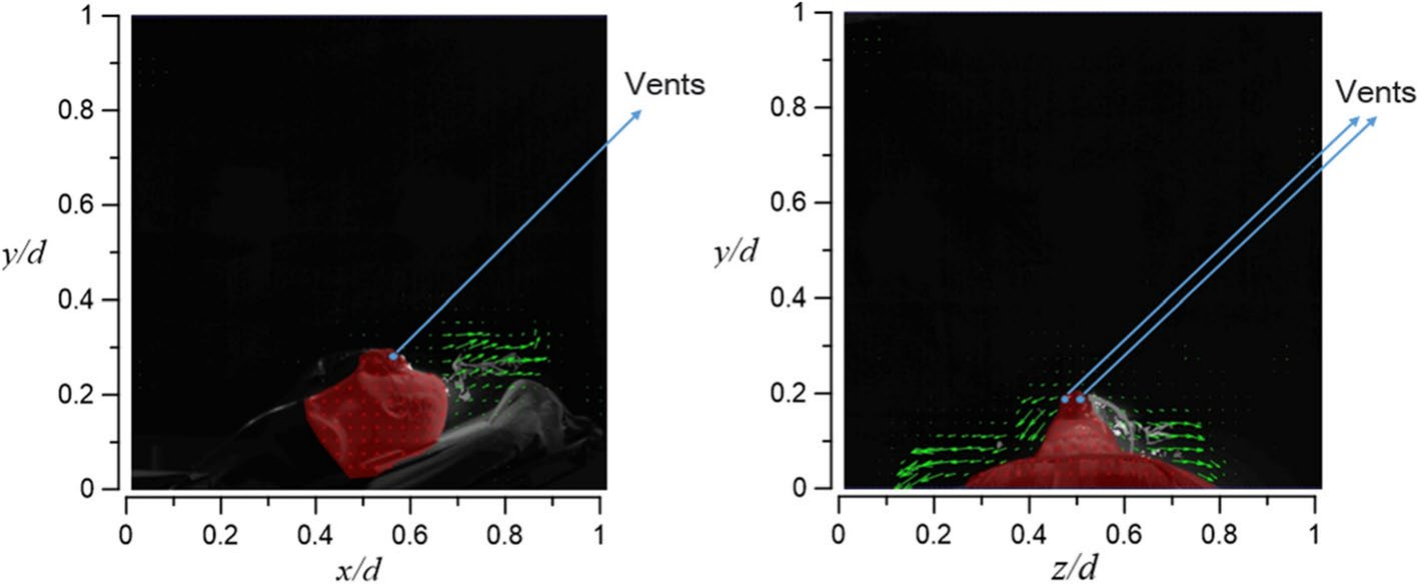
Flow field visualization result of case 4.2 (left: side view; right: face view)

Unlike the previously discussed oxygen supply equipment, NRM has two vents, one on each side of the nose bridge. Due to this reason, in addition to leaking from the gap between the oxygen mask and the face, the exhaled smoke also diffuses from the two vents to the head. As observed in Fig. 31, the smoke flows to both sides of the dummy (approximately axial level of 0.35 < *z*/*d* < 0.8) in both case 4.1 and case 4.2, in which the dummy’s tidal volume increases from 10 to 20 l/min, and the oxygen supply rate is fixed at 15 l/min. It was observed that the smoke barely flows upward. Similar results were observed for case 4.2 shown in Fig. 32. The only difference between the results of case 4.1 and case 4.2 is that the larger amount of smoke appears on both sides of the face after increasing the tidal volume.

#### Continuous positive airway pressure (CPAP)

**Fig. 33:**
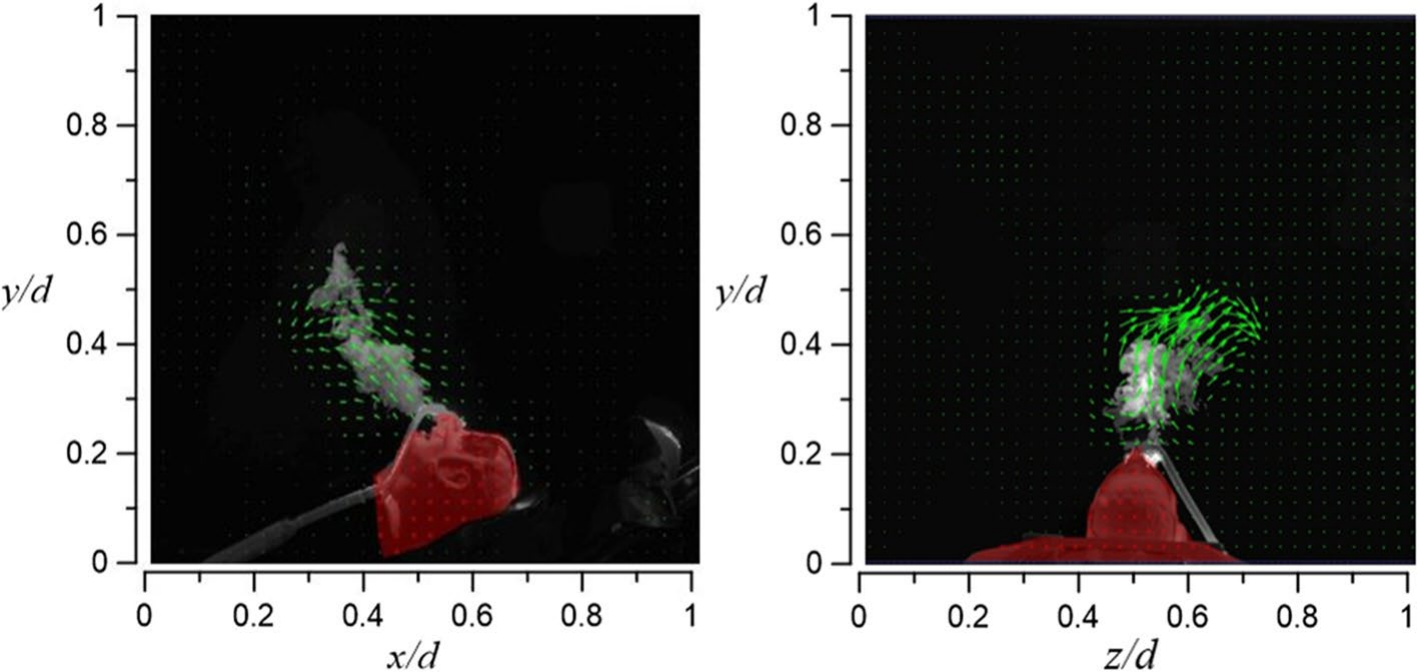
Flow field visualization result of case 5.1 (left: side view; right: face view)

**Fig. 34:**
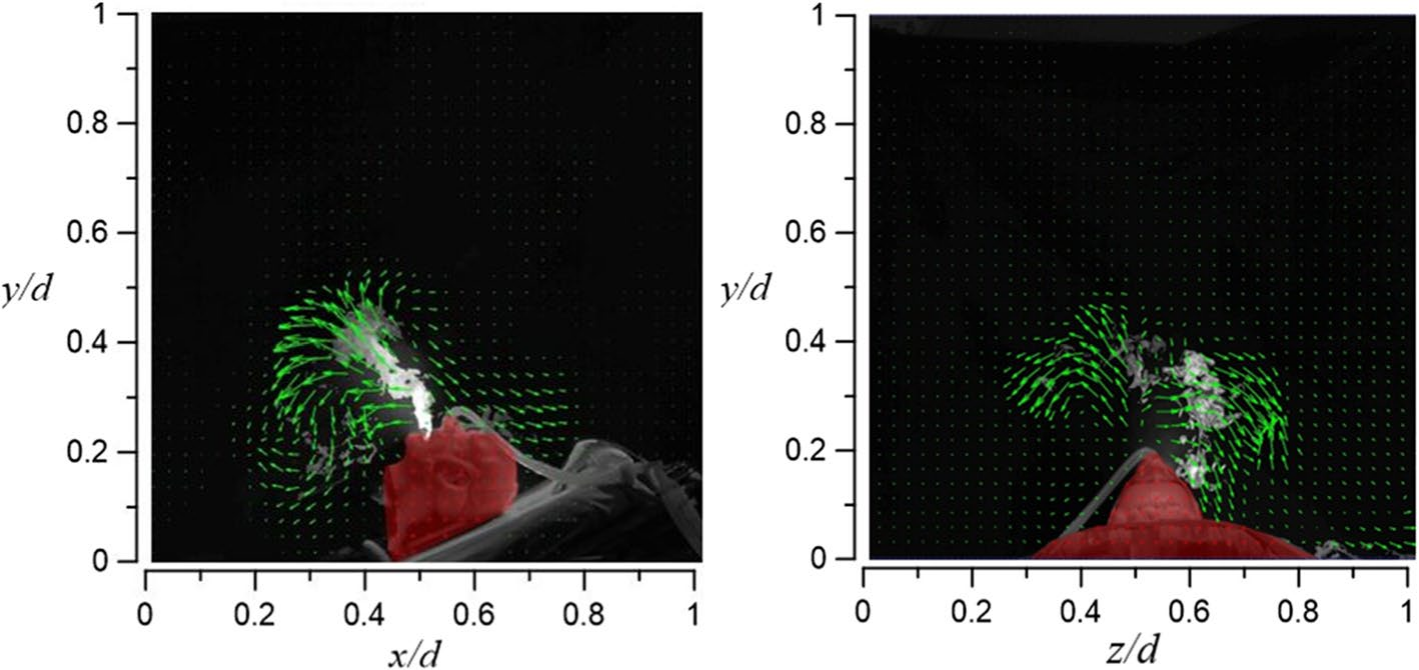
Flow field visualization result of case 5.2 (left: side view; right: face view)

Figure 33 shows the flow visualization results for case 5.1. The smoke can rise up to a vertical level of *y*/*d* ≈ 0.65 while slightly bending to the right side at an axial level of 0.5 < *z*/*d* < 0.7. Figure 34 shows the flow visualization for case 5.2. The smoke can rise up to a vertical level of *y*/*d* ≈ 0.5 while the smoke is distributed to both sides of the dummy at an axial level of 0.2 < *z*/*d* < 0.8.

Figures 33 and 34 show the visualizations of CAPA’s oxygen supply rate of 40 l/min. Due to this high rate of supply of oxygen, the smoke diffusion height can reach up to 36 cm. Raising the tidal volume from 10 l/min (case 5.1) to 20 l/min (case 5.2) is the primary reason that the smoke starts to flow to the left side of the dummy.

#### Ventilator-assisted pre-oxygenation (VAPOX)

**Fig. 35:**
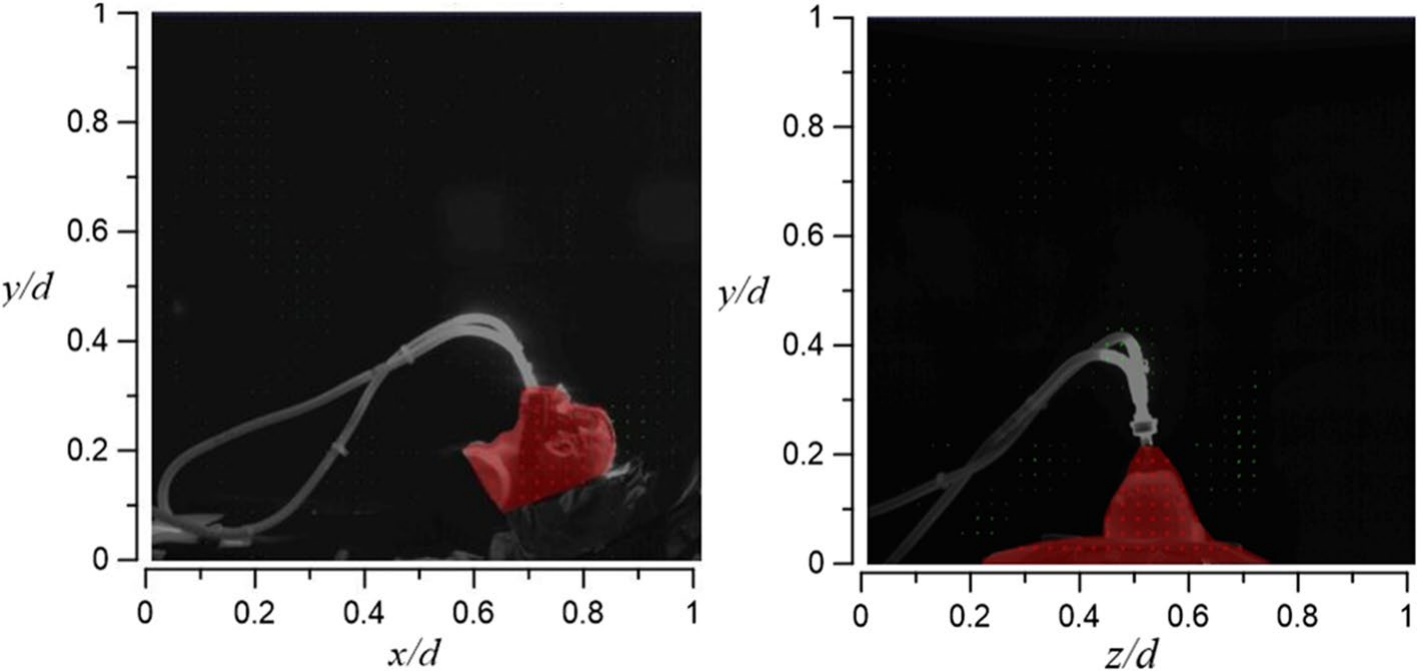
Flow field visualization result of case 6.1 (left: side view; right: face view)

**Fig. 36:**
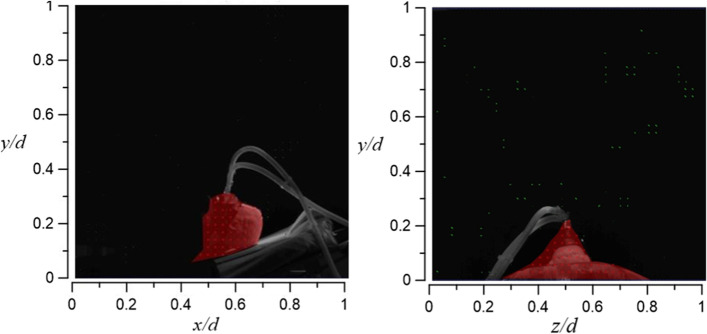
Flow field visualization result of case 6.2 (left: side view; right: face view)

The oxygen supply rate of VAPOX is 28 l/min, which is the fourth-highest in the studied cases. For other methods, such high flow rates may well be expected to result in higher leak rates from the mask to the face seal; however, the VAPOX system has the advantage of excellent airtightness. When it fits the patient’s face completely, the smoke rarely spreads to the environment, resulting in almost no smoke in the flow field visualization (Figs. 35 and 36). Even with increases in the dummy’s tidal volume from 10 l/min (case 6.1) to 20 l/min (case 6.2), there is barely any smoke in the visualization of the flow field, as shown in Figs. 35 and 36. Hence, the PAO concentration in case 6 is speculated to be the lowest amount for all tests (Table 2).

**Table 2:**
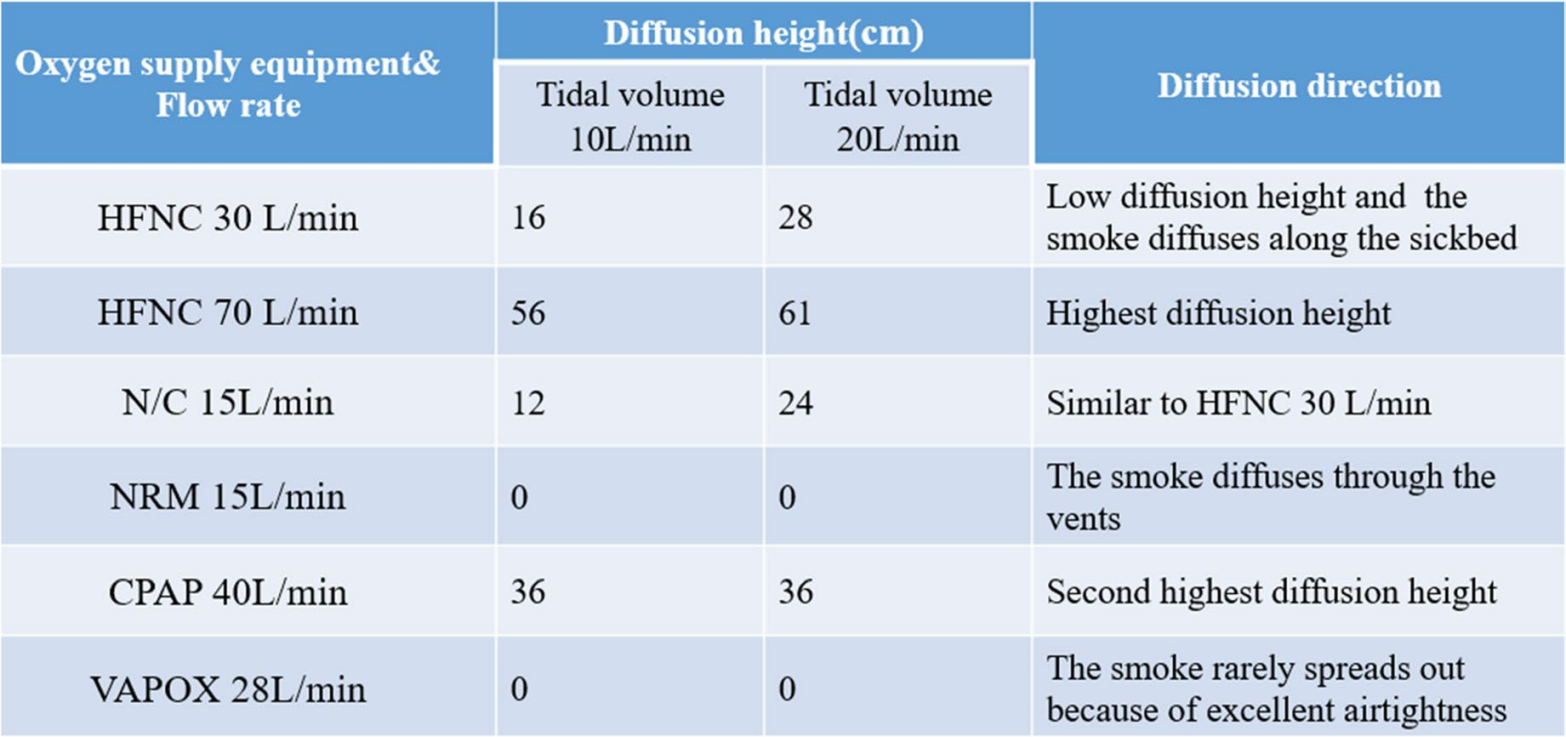
Measured diffusion height and direction for different oxygen supply equipment and flow rates

The diffusion height is positively correlated to the oxygen supply, and the structure of the oxygen supply equipment will also affect the diffusion direction of the exhaled airflow. For example, NRM and VAPOX cover the mouth and nose, but NRM is not as airtight as VAPOX, and there are vents above the nose bridge, resulting in the diffusion of smoke through the vents and the gap between the mask and face.

### Aerosol diffused concentration

#### Concentration at each measurement point

The result of aerosol diffused concentration (data presented as the ratio between downstream concentration and upstream concentration) is shown in Table 4. Apart from NRM, all the oxygen supply equipment has a foot side concentration greater than the head side. The possible explanation for this is the exhalation port of the dummy is facing the foot side, making the foot side with the highest concentration. Alternatively, NRM has the vents facing the head, which makes the head side with a higher concentration. In the flow field visualization when using VAPOX, the tracked gas is unable to be seen in the resulting chart; moreover, it has a relatively low diffused concentration. The other finding is that when comparing values in Tables 3 and 4, the measured concentration of the dummy having a tidal volume of 20 l/min is always greater than that of having 10 l/min, resulting from the higher amount of aerosol induced by the larger expiratory flow. The following table shows the relative percentages of PAO concentration produced at downstream and upstream measurement points.

**Table 3:** PAO concentration at each point under tidal volume 10 l/min

Concentration(%)	HFNC 30	HFNC 70	N/C	NRM	CPAP	VAPOX
**Head**	0.0571%	0.0506%	0.0328%	0.0443%	0.0327%	0.0105%
**Body**	0.0539%	0.0534%	0.0338%	0.0156%	0.0284%	0.0129%
**Foot**	0.1047%	0.1188%	0.1067%	0.0305%	0.142%	0.0272%

**Table 4:** PAO concentration at each point under tidal volume 20 l/min

Concentration(%)	HFNC 30	HFNC 70	N/C	NRM	CPAP	VAPOX
**Head**	0.0694%	0.0635%	0.0513%	0.0595%	0.1012%	0.0284%
**Body**	0.0563%	0.0704%	0.0443%	0.0241%	0.0719%	0.0193%
**Foot**	0.1175%	0.1751%	0.1421%	0.0478%	0.1773%	0.0358%

#### Correlation between aerosol concentration and time

**Fig. 37:**
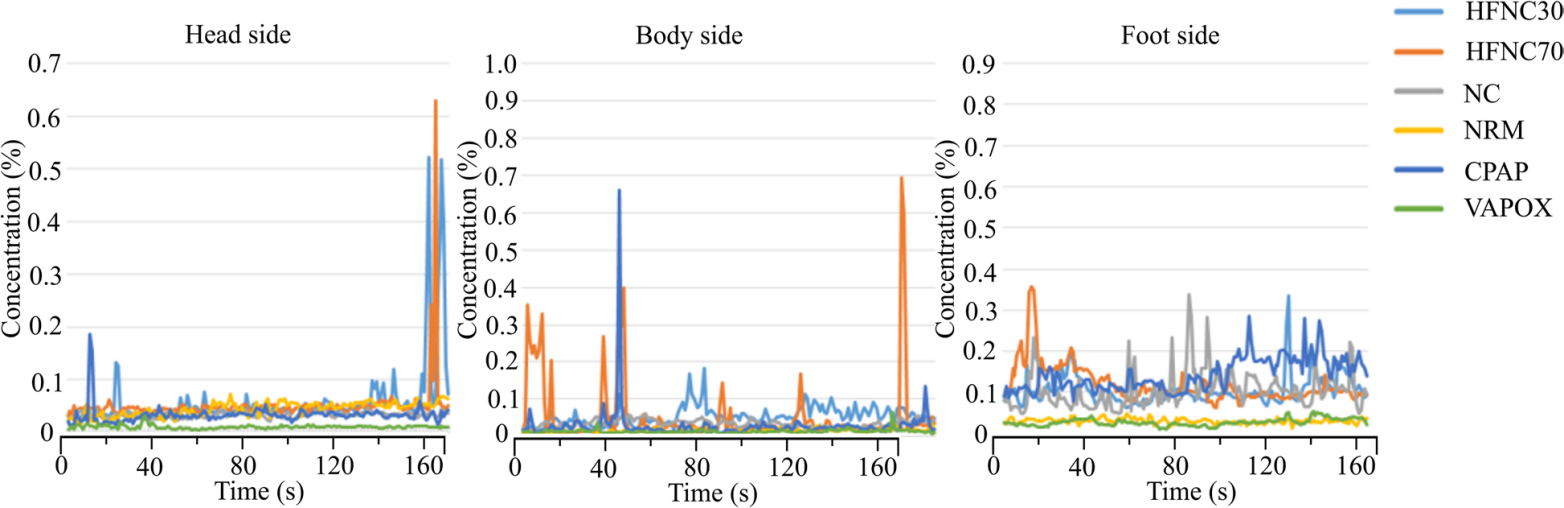
Aerosol concentration variation at tidal volume 10 l/min

**Fig. 38:**
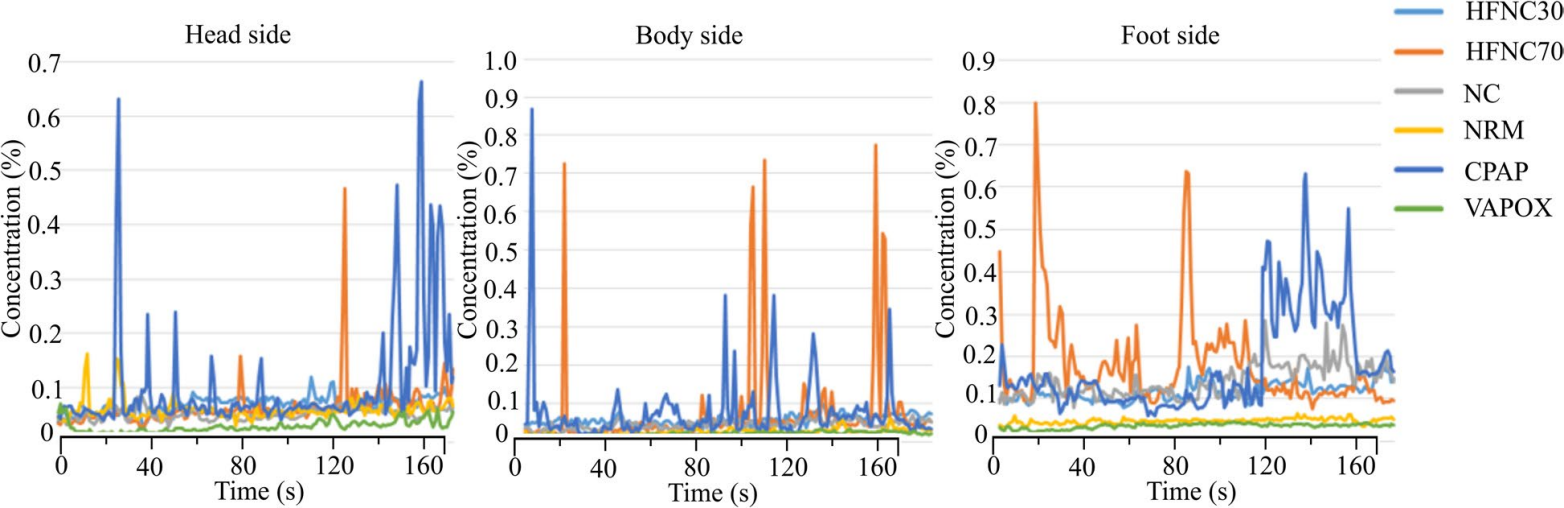
Aerosol concentration variation at tidal volume 20 l/min

Figures 37 and 38 show the aerosol concentration variations at different tidal volumes of 10 l/min and 20 l/min, respectively. Different aerosol types (HFNC 30, HFNC 70, N/C, NRM, CPAP, and VAPOX) and various probe positions (of the head side, body side, and foot side) were examined. Comparing the two tidal volume cases (comparison of Figs. 37 and 38), it is found that there are more concentration peaks when the tidal volume is at 20 l/min. The reason for this is probably because the smoke accumulates in the dummy’s mouth and produces a cloud of smoke when exhaling. If the surrounding flow field does not dilute the smoke, the concentration will rise. The aerosols HFNC at 70 and CPAP have a sharp rise in concentration at each point. It is presumed that the greater the flow rate of the oxygen supply device, the more likely it is to cause a sharp rise in the concentration.

## Conclusion

In this study, the flow distribution of a large area in an operating room was examined by smoke flow visualization and PIV to evaluate the PIC effectively. Moreover, the results were verified by the PAO concentration test. The following findings were observed during the experiments.

The greater the tidal volume of the patient, the higher the risk for medical staff.

From the result of HFNC70 and CPAP, it can be concluded that the larger the oxygen supply rate is, the farther the distance and range of aerosol diffusion.

Apart from NRM, the foot side concentration is the highest when using other oxygen supply devices.

Since NRM has the vents facing the head, the aerosol concentration on the head side is higher than on the trunk side and the foot side.

If the patient has a lower oxygen demand (less than 15 l/min), NRM can generate lower aerosol diffusion compared with a nasal cannula.

Among all the studied devices, VAPOX has the advantage of isolating the patient’s nose and mouth, making the aerosol difficult to diffuse to the environment. Hence, the detected aerosol concentration is lower when using VAPOX.

The detected aerosol concentration under different devices is HFNC70 > CPAP > HFNC30 > Nasal cannula > NRM > VAPOX.

## Future work

Six different oxygen supply devices were examined to analyze the infection risk to medical personnel when performing intubation procedures. The diffusion of the infective agent could originate through the nose and mouth or the gap between the mask and the face. Hence, to prevent the infective agent from diffusing to the surrounding area and infecting the medical staff, a possible solution is to design a *transparent mask*. Such a solution could cover the patient’s face during positioning and collocate with exhaust equipment to create a small negative pressure volume. In this case, the escape of the infective agent into the wider environment can be avoided as it is exhaled from the patient.
